# Compression and Interpenetration of Ionic Microgels in Electrostatically Self-Assembled Clusters

**DOI:** 10.1021/acsnano.6c00724

**Published:** 2026-07-24

**Authors:** Alexander V. Petrunin, Hannah F. Mathews, Tom Höfken, Philipp Leclercq, Ralf Schweins, Pablo Mota-Santiago, Stefanie Schneider, Andrij Pich, Andrea Scotti

**Affiliations:** † Institute of Experimental Physics of Condensed Matter, Heinrich-Heine University, Universitätsstr. 1, Düsseldorf 40225, Germany; ‡ Institute of Physical Chemistry, RWTH Aachen University, Landoltweg 2, Aachen 52074, Germany; § DWI - Leibniz Institute for Interactive Materials e. V., Forckenbeckstr. 50, Aachen 52074, Germany; ∥ Institute of Technical and Macromolecular Chemistry, RWTH Aachen University, Worringerweg 2, Aachen 52074, Germany; ⊥ 56053 Institut Laue-Langevin ILL, DS/LSS, 71 Avenue des Martyrs, Grenoble F-38000, France; # Australian Synchrotron, ANSTO, Clayton, Victoria 3168, Australia; ¶ MAX IV Laboratory, 5193 Lund University, Lund 22100, Sweden; ∇ Aachen Maastricht Institute for Biobased Materials (AMIBM), Brightlands Chemelot Campus, Maastricht University, Geleen 6167 RD, The Netherlands; ○ Division of Physical Chemistry, 5193 Lund University, Lund 22100, Sweden

**Keywords:** self-assembly, microgels, SANS, SAXS, molecular dynamics

## Abstract

Colloidal self-assembly is a powerful strategy to obtain materials with desired softness, porosity and biocompatibility. When soft building blocks are used, these properties can change dramatically, compared to materials produced using hard particles. However, the fate of individual colloids inside these assemblies is not fully understood. Here, asymmetric mixtures of soft microgels with opposite electrical charges are used as model building blocks to assemble colloidal clusters. The changes in the form factor of individual microgels due to cluster formation are analyzed using small-angle neutron scattering with contrast variation, complemented with small-angle X-ray scattering and molecular dynamics simulations. A strong compression of the fuzzy shell of a microgel is observed, which results in a peculiar core–shell architecture of the microgel. In contrast, a reference system of similarly charged microgels shows osmotic deswelling in both the dense core and fuzzy shell, as expected. Molecular dynamics simulations reveal that the polymer density at contact between the oppositely charged microgels increases due to the entropically favorable counterion release. To maximize the number of released counterions, the microgels compress their shells or interpenetrate each other. The results show that the structure of individual soft colloids may be significantly altered during the assembly of clusters, which can influence the larger hierarchical assembly.

## Graphical abstract

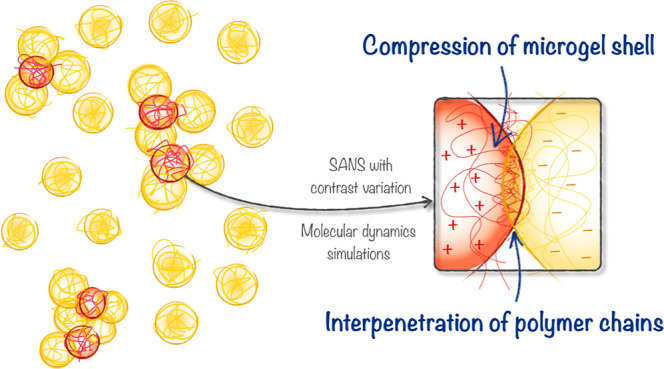

Colloidal suspensions can form various ordered or disordered phases that bear many similarities with atomic and molecular systems. This makes colloids a powerful tool to study phase transitions^1–4^ and a versatile platform to assemble new functional materials with predefined structure–property relationships.^5–7^ The simplest case of colloidal self-assembly is clustering in the presence of an attractive interaction potential. This phenomenon is common in many types of colloids, ranging from hard spheres with short-range attraction^8–10^ to concentrated protein or antibody solutions.^11,12^ Especially for antibody solutions, cluster formation plays a critical role because it dramatically increases their viscosity, which complicates the development of pharmaceutical formulations.^13,14^

At high volume fractions, attractive interaction between particles can lead to cluster percolation and formation of weak viscoelastic solids known as “colloidal gels”.^15,16^ They are commonly found in foods, cosmetics, coatings and other everyday materials.^17,18^ These gels, as well as protein aggregates, consist of soft building blocks, such as biopolymer particles or emulsion droplets that cannot be described as simple hard spheres.^19^ From a materials science perspective, using soft building blocks for self-assembled materials offers a huge potential to control material biocompatibility,^20–22^ microstructure^7,23,24^ or introduce stimuli-responsiveness.^7,25,26^

The softness of the building blocks can dramatically affect the microstructure and functionality of the self-assembled material. In the case of aggregation by depletion interactions, particle softness leads to formation of dense clusters with fractal dimensions higher than what is predicted by the diffusion-limited cluster aggregation (DLCA) model.^27,28^ This is due to the capacity of soft particles to effectively overlap via deformation or interpenetration and their faster aggregation kinetics.^27^ However, when the aggregation is driven by electrostatic attraction between oppositely charged species, the picture becomes more complicated. On the one hand, dense clusters can be favored because they allow the particles to maximize the contact area and mutually neutralize charges.^24,29^ On the other hand, electrostatic repulsion between similarly charged particles can prevent the dense packing of clusters, favoring more porous structures.^23,24,30,31^ To reconcile this disagreement, it is crucial to know how individual particles in these assemblies behave, including any changes in their size, shape or charge that occur as a result of cluster formation.

To answer this question, microgels are one of the most suitable model system among synthetic soft colloids. Microgels are colloidal polymer networks swollen in a good solvent. They can be synthesized controlling their size, internal structure, compressibility and electrical charge with an exceptionally high degree of precision compared other soft colloids.^32–36^ Microgels have been widely used for bottom-up assembly of functional and stimuli-responsive materials.^37,38^ Examples of such materials include well-defined colloidal clusters (“colloidal molecules”),^39–45^ colloidosomes or microcapsules,^46–49^ responsive heteroaggregates,^50,51^ interfacial films,^52,53^ responsive colloidal crystals^26,54–56^ or colloidal gels.^7,25,26,45^ Furthermore, binary dense phases like microgel-polyelectrolyte gels,^57,58^ binary attractive glasses^59^ and coacervates from oppositely charged microgels^60–62^ have attracted significant attention from both fundamental and applied perspectives.

The supracolloidal assembly of microgels typically occurs via electrostatic attraction, hydrophobic interaction or chemical cross-linking. Among these strategies, electrostatic attraction is often the method of choice due to its robustness and tunability via changes of the solution’s pH or ionic strength.^41,45,63^ Electrostatics also play a crucial role in protein and antibody aggregation,^64–67^ so microgel assemblies can be considered a simplified but helpful model system to shed light on the changes of individual proteins’ architecture and how these changes affect the aggregate’s final properties.^19^

The size and structure of individual microgels within concentrated assemblies can be determined using small-angle neutron scattering with contrast variation (CV-SANS). In this method, the scattering from a few hydrogenated “tracer” particles is measured in a matrix of partially deuterated particles that are contrast-matched by a H_2_O/D_2_O solvent mixture.^68–70^ CV-SANS has been pivotal to study the response of microgels to crowding in suspensions with purely repulsive interactions (e.g., fluids, crystals, glasses), where isotropic deswelling,^68–72^ anisotropic deformation (faceting)^70,73,74^ and interpenetration^75,76^ have been reported. Now, the same approach can be used in microgel assemblies with attractive interactions, such as clusters, gels or coacervates, where even stronger effects are possible. Already in dilute suspension, complexation of microgels with polyelectrolytes or charged nanoparticles leads to strong deswelling^63,77–79^ and changes in internal structure.^80,81^ Yet, how this behavior translates to mixtures of oppositely charged microgels and their supracolloidal assemblies is not known.

Here, we report how individual ionic microgels behave inside electrostatic colloidal clusters combining CV-SANS and molecular dynamics simulations. The clusters are formed spontaneously upon mixing the oppositely charged microgel species at low ionic strength and highly asymmetric composition. The formed clusters are characterized using small-angle X-ray scattering (SAXS), light scattering and shear rheology. We vary the effective volume fraction at which the clusters are obtained to explore the interplay between electrostatics and crowding. For comparison, suspensions of identical similarly charged microgels are studied in parallel. Using CV-SANS, we observe strong compression of the microgels inside the clusters, which differs markedly from the deswelling in similarly charged microgel suspensions. Molecular dynamics simulations provide crucial information on charge compensation and counterion release, which explain this peculiar behavior. Overall, the results suggest that charge regulation in the cluster plays a more important role than osmotic or steric effects, in contrast to what was observed for suspensions of purely repulsive microgels.

## Results and Discussion

### Microgel Characterization in Dilute Suspensions

To prepare the microgel mixtures with opposite or similar charges, we synthesized three batches of poly­(*N*-isopropylacrylamide) (pNIPAm) microgels: (i) negatively charged deuterated microgels (D7-pNIPAm), referred to as D_–_; (ii) positively charged protonated microgels, H_+_; and (iii) negatively charged protonated microgels, H_–_. The deuterated microgels are necessary to perform contrast variation in the SANS experiments (see below). The synthesized microgels are weakly cross-linked to make them sufficiently soft: we used 2 mol % *N*,*N*′-methylenebis­(acrylamide) (BIS) cross-linker for H_+_ and H_–_ microgels, but 3 mol % BIS for D_–_ microgels to compensate for the absence of self-cross-linking of the deuterated monomer (D7-NIPAm).^33^ We incorporated 10 mol % of ionizable comonomer (*N*-(3-aminopropyl)­methacrylamide hydrochloride (APMH) for H_+_ or methacrylic acid (MAAc) for H_–_/D_–_) during synthesis to provide a high charge density to the particles. The distribution of charges across the polymer network is expected to be approximately homogeneous due to the delayed addition of MAAc in the synthesis of H_–_ and D_–_ microgels (3 min after initiation; see Experimental section) and similar copolymerization rates of APMH and NIPAm.^82^ Using FT-IR and ^1^H NMR spectroscopy as described in the literature,^61^ we estimated the real comonomer content after purification to be 9.5 mol % in H_+_ microgels, 8.5 mol % in H_–_ microgels, 10.8 mol % in D_–_ microgels. The FT-IR and ^1^H NMR spectra are shown in Supporting Information, Figures S1 and S2, respectively. The lower amounts of ionizable comonomers in H_+_ and H_–_ microgel samples can be explained by their high water solubility and accumulation in the non-cross-linked polymer fraction, which was removed during the dialysis step. For an estimation of the molecular weights of the microgels, see Section S3 (Supporting Information).

For all experiments shown in this work, the microgels were redispersed at *T* = 20 °C in a 10 mM phosphate buffer solution at pH 7.0 to ensure that they are fully swollen, and both the APMH and MAAc groups are fully ionized. Under these conditions, the electrophoretic mobilities of the microgels, μ, are 0.83 ± 0.01, −1.80 ± 0.03 and −1.89 ± 0.03 μm·cm/(V·s) for H_+_, H_–_ and D_–_microgels, respectively.

We determined the hydrodynamic radii of the microgels, *R*_h_, under these conditions using multiangle dynamic light scattering (DLS). We also analyzed their size, internal structure and polydispersity by measuring the form factors in dilute suspension using SANS or SAXS and fitting the data to the fuzzy-sphere model, Figure S3 (Supporting Information). For D_–_ microgels, a relatively concentrated sample (ζ = 0.42 ± 0.04) was used for this purpose. Due to the presence of a structural peak, the fitting was performed at *q* > 5 × 10^–2^ nm^–1^, where *S*(*q*) ≈ 1. A sample at a lower ζ showed featureless scattering curves in the middle *q*-range in both SANS and SAXS due to the low contrast of these very soft particles, Figure S4, not allowing the form factor fits to converge to physically reasonable parameters. Fitting the D_–_ form factor in a more concentrated suspension also allows us to partially compensate for any deswelling effects when obtaining the effective structure factors of the mixtures (see below).

Table 1 shows the hydrodynamic radius *R*_h_, as well as the parameters obtained from the form factor fits: total radius *R*, the radius of the core *R*_c_, the width of the fuzzy shell 2σ_out_ and polydispersity *p*. Although the *R*_h_-values of the three microgels are very similar (≈115 nm), the analysis of their form factors reveals subtle differences between these particles. Negatively charged microgels, H_–_ and D_–_, have significantly smaller fuzzy-sphere radii *R* than their *R*_h_, by 30 and 71 nm, respectively. This discrepancy is typically attributed to the dangling polymer chains at the periphery of the microgel, which contribute only to the hydrodynamic radius, but are invisible in static scattering.^83^ Indeed, it was previously shown that pNIPAm microgels copolymerized with MAAc can have unusually long dangling chains at low ionic strengths, from a few tens to a few hundreds of nanometers.^84,85^ The D_–_ microgels also have a relatively large meshsize of the polymer network, ξ = 10 ± 1 nm, indicating a large contribution of the long chains to overall network structure. Conversely, for H_+_ microgels, the dangling chains are rather compact and extend by *R*_h_ – *R* = 17 nm. The internal structure of H_+_ microgels is also less fuzzy compared to the H_–_ and D_–_ microgels: the relative width of the fuzzy shell, 2σ_out_/*R*, is 42% for H_+_ vs 51 and 65% for H_–_ and D_–_, respectively. The polydispersity of H_+_ microgels is also lower compared to H_–_ and D_–_: (16 ± 2)% vs (25 ± 3)% and (28 ± 3)%. Therefore, the positively- and negatively charged microgels that we use in this study have comparable hydrodynamic size and charge density, but slightly different internal structure and size polydispersity. Such differences are hard to avoid in copolymer and deuterated microgels and should be taken into account when interpreting their behavior in the assemblies or dense suspensions. However, the use of the tracing method in CV-SANS experiments (as opposed to the zero-average contrast (ZAC) approach^75,84^ ) makes the differences between the deuterated and protonated particles insignificant for an accurate form factor measurement. This is because deuterated particles do not contribute to the coherent scattering signal in CV-SANS, but are used as a passive crowding agent.^68,69,86^

**Table 1:** Hydrodynamic Radii *R*_h_, Electrophoretic Mobilities μ, Parameters of the Form Factor Fits, Including Radius of the Core *R*_c_, Width of the Fuzzy Shell 2σ, Meshsize ξ and Polydispersity *p*, as well as Viscometry Conversion Constants *k* for the Studied Microgels

microgel	*R*_h_ [nm]	μ [μm·cm/(V·s)]	*R* [nm]	*R*_c_ [nm]	2σ_out_ [nm]	ξ [nm]	*p* [%]	*k*
H_–_	113 ± 2	–1.80 ± 0.03	83 ± 4	41 ± 3	42 ± 3	5 ± 1	25 ± 3	64.5 ± 1.3
H_+_	118 ± 2	0.83 ± 0.01	101 ± 5	59 ± 3	42 ± 3	4 ± 1	16 ± 2	31.3 ± 1.3
D_–_	117 ± 1	–1.89 ± 0.03	46 ± 2	16 ± 1	30 ± 2	10 ± 1	28 ± 3	136 ± 12

### Cluster Formation between Oppositely Charged Microgels

To prepare the self-assembled clusters, we mixed a few H_+_ microgels with a large excess of D_–_ microgels (oppositely charged mixtures) using a syringe pump (see Experimental Section). In this way, we avoided bulk heteroaggregation of oppositely charged particles and phase separation of the coagulate. The mixed H_+_/D_–_ suspensions were slightly more turbid than individual stock solutions, but showed no sedimentation for over 6 months at room temperature. As a reference system, we prepared the mixtures of a few H_–_ microgels in the matrix of D_–_ microgels (similarly charged mixtures) by simply mixing the two stock solutions with a pipette. The concentration of particles is expressed using the generalized volume fraction, ζ, which we determine from viscosimetry measurements, Figure S5 and Section S3 (Supporting Information). ζ is commonly used in the microgel literature^71,87,88^ and corresponds to the volume fraction that the particles would occupy in their fully swollen state, i.e. it can exceed 1 in overpacked suspensions. For the binary mixtures, we kept the generalized volume fraction of protonated microgels (H_+_ or H_–_), ζ_H_, low and almost constant: between 0.040 ± 0.004 and 0.090 ± 0.008. Instead, we varied the generalized volume fraction of deuterated microgels, ζ_D_, to cover a broad ζ-range of the mixtures, where ζ = ζ_H_ + ζ_D_ values are between 0.31 ± 0.02 and 3.06 ± 0.02. The volume fraction ratio of the negatively charged D_–_ microgels to positively charged H_+_ microgels, ζ_D_/ζ_H_, was between ≈7:1 in the least concentrated sample and ≈49:1 in the most concentrated one. Thus, the obtained mixtures had a highly asymmetric composition. All samples were prepared in the 80 wt % D_2_O/20 wt % H_2_O solvent mixture (incl. 10 mM phosphate buffer, pH 7.0) for contrast-matching in SANS, so that the *exact same* samples are characterized using CV-SANS and other methods.

First, we probed the structure of the H_+_/D_–_ mixtures using small-angle X-ray scattering (SAXS). The plots of the SAXS intensity, *I*(*q*), vs scattering vector *q* are shown in Figure S6 (Supporting Information). Since both protonated and deuterated particles contribute to the signal in the *I*(*q*), we can extract the effective structure factor of the system, *S*_eff_(*q*), as follows^74,89^

1 $$S_{eff}\left(q\right)≃\frac{I\left(q\right)}{n_{H}P_{H}\left(q\right)+n_{D}P_{D}\left(q\right)}$$

where *n*_H_, *n*_D_ and *P*_H_(*q*), *P*_D_(*q*) are the number densities and form factors of the protonated (H) and deuterated (D) microgels, respectively.

Figure 1A shows the *S*_eff_(*q*) vs scattering vector *q* for the H_+_/D_–_ mixtures with increasing ζ (from bottom to top; the data are shifted along *y*-axis for clarity). The curves have two distinct features: an upturn at *q* < 3 × 10^–2^ nm^–1^, which is independent of ζ, and a structural peak at *q* ≈ 5 × 10^–2^ nm^–1^, which grows higher with increasing ζ. The low-*q* upturn is a typical signature of attractive systems, indicating formation of irregular colloidal clusters or aggregates.^14,90^ By looking at the power-law scaling of the upturn, the fractal dimension of the aggregate, *d*_f_, can be determined^90–92^

2 $$I\left(q\right)\propto S_{eff}\left(q\right)\propto {\left(1+\frac{2R_{g}^{2}q^{2}}{3d_{f}}\right)}^{-d_{f}/2}$$

where *R*_g_ is the average radius of gyration of the aggregates.

**Figure 1:**
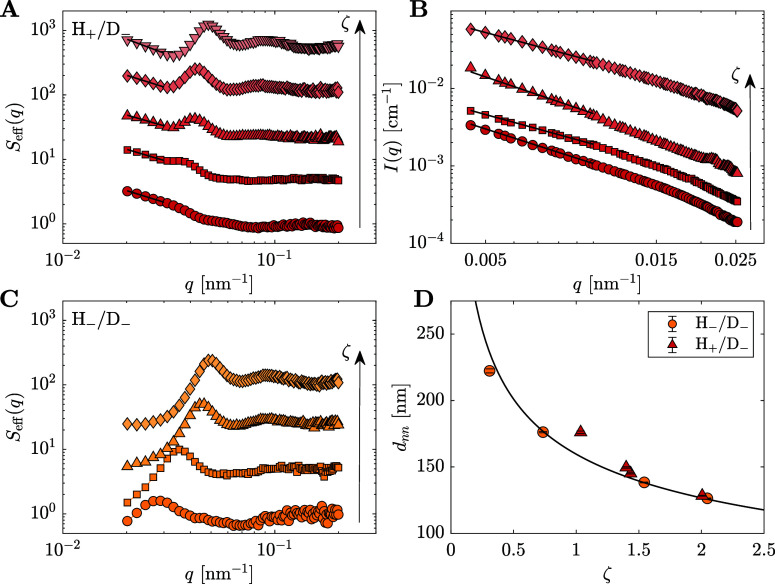
(A) Effective structure factors from SAXS *S*_eff_(*q*) vs scattering vector *q* for H_+_/D_–_ mixtures at ζ = 0.76 ± 0.06 ζ = 1.04 ± 0.09, ζ = 1.4 ± 0.1, ζ = 2.0 ± 0.2 (from bottom to top). Solid lines correspond to fits using eq 3. (B) SLS intensities vs scattering vector *q* for H_+_/D_–_ mixtures diluted from ζ = 0.33 ± 0.03, 0.74 ± 0.07, 1.4 ± 0.1, 2.0 ± 0.2 (from bottom to top). Solid lines correspond to fits using eq 3. (C) Effective structure factors from SAXS *S*_eff_(*q*) vs scattering vector *q* for H_–_/D_–_ mixtures at ζ = 0.31 ± 0.03, ζ = 0.74 ± 0.06, ζ = 1.5 ± 0.1, ζ = 2.1 ± 0.2 (from bottom to top). (D) Nearest-neighbor distance *d*_nn_ in H_+_/D_–_ mixtures (red upward triangles) and in H_–_/D_–_ mixtures (yellow circles). Solid line corresponds to the fit of the H_–_/D_–_ data using eq 4.

For our experimental *q*-range, *R*_g_^2^ *q* ^2^ ≫ 3*d*_f_/2, so eq 2 simplifies to

3 $$I\left(q\right)\propto S_{eff}\left(q\right)\propto q^{-d_{f}}$$

By fitting the low-*q* regime of the structure factors, Figure 1 (A, black solid lines), we find *d*_f_ gradually increases from 0.90 ± 0.03 at ζ = 0.76 ± 0.06 to 1.23 ± 0.06 at ζ = 2.0 ± 0.2. These values (*d*_f_ ≈ 1) are consistent with the presence of rod-like structures.^90^ A similar fit to the *I*(*q*) SAXS data yields *d*_f_ values between 1.7 ± 0.1 and 1.93 ± 0.08, Figure S6 (B, black solid lines). However, the low-*q* power-law regime partly overlaps with the structural peak, possibly distorting the scaling.

To eliminate the influence of the peak, we diluted selected H_+_/D_–_ mixtures with a large excess (>100*x*, v/v) of buffer and analyzed them using static light scattering (SLS). Figure 1B shows the SLS intensity *vs q* with increasing ζ, which the samples had before dilution (from bottom to top). By fitting the SLS data at *q* < 1 × 10^–2^ nm^–1^ with eq 3, we obtain fractal dimensions between *d*_f_ = 1.23 ± 0.05 and 1.6 ± 0.1, which change irregularly with increasing ζ. Such low values correspond to branched structures, in between rod-like aggregates, *d*_f_ = 1,^90^ and aggregates predicted by the diffusion-limited cluster aggregation theory (DLCA), *d*_f_ = 1.7–1.8.^93^ The open morphology of the clusters can be explained by the electrostatic repulsion between similarly charged microgels, which prevents them from packing densely.

We further confirmed the formation of stable clusters using multiangle dynamic light scattering (DLS). The angle-dependent decay rates Γ of the DLS intermediate scattering functions showed a linear Γ vs *q* ^2^ dependence, Figure S7, allowing us to extract the effective hydrodynamic radii, *R*_h_. The obtained *R*_h_ values for the H_+_/D_–_ mixtures after dilution (>100*x*, v/v) are significantly larger than the *R*_h_ of individual microgels, Figure S8, and correspond to the average values between clusters and free microgels present in the samples.

In contrast to the H_+_/D_–_ system, the structure factors of the H_–_/D_–_ mixtures show no upturn at low *q*-values, Figure 1C. Instead, the value of the *S*_eff_(*q*) tends to a plateau ≈0.2 when *q*→0, Figure S9, indicating the absence of any clustering in these mixtures.

The structural peak in the *S*_eff_(*q*) of both H_+_/D_–_ and H_–_/D_–_ mixtures shifts to higher *q*-values with increasing ζ. For H_+_/D_–_, it first appears as a shoulder at ζ = 0.76 ± 0.06 (circles in Figure 1A) but then grows in magnitude with increasing ζ. The peak position, *q*_max_, is related to the nearest-neighbor distance between the microgels, *d*_nn_, as *d*_nn_ = 2π/*q*_max_.^71^ Figure 1D shows the *d*_nn_ values obtained from the peaks as a function of ζ for the H_+_/D_–_ mixtures (red upward triangles) and H_–_/D_–_ mixtures (yellow circles). In both cases, the peak position decreases with increasing ζ, following a well-known scaling law for particles interacting via a soft repulsive potential (black solid line)^71,84,94,95^

4 $$d_{nn}\propto \zeta^{-1/3}$$

This proves that the peak originates from purely repulsive interactions between the vast majority of similarly charged D_–_ microgels in the H_+_/D_–_ mixtures, or between both H_–_ and D_–_ microgels in the H_–_/D_–_ mixtures. The *d*_nn_ values are similar in the two mixtures at ζ ≥ 1.5, whereas at ζ < 1.5 the values for H_+_/D_–_ are slightly higher compared to the H_–_/D_–_. At ζ = 0.76 ± 0.06 the peak is almost absent in the H_+_/D_–_ system. The increase of the *d*_nn_ values, followed by the disappearance of the peak at low ζ, is likely the result of charge neutralization inside the clusters, which leads to lower electrostatic repulsion between the microgels or clusters and less swelling (lower “true” volume fraction) of individual microgels. Formation of compact clusters creates additional free volume, so the *d*_nn_ slightly increases.

In order to gain a better understanding of the microstructure of the mixtures, molecular dynamics simulations were performed on a small number of charged model microgel networks incorporating explicit counterions. We compare two systems: the first contains positively and negatively charged microgels in a 1:12 ratio ((+/−), similar to H_+_/D_–_); the second consists only of negatively charged microgels ((−/−), similar to H_–_/D_–_). The ratio of 1:12 is chosen within the range of ζ_D_/ζ_H_ used in experiments, but is kept constant to identify the influence of crowding (ζ) on the clustering, which is harder to achieve in experiments. Although we could not reproduce the actual size and fractal nature of the clusters in simulation due to a limited size of the simulation box, we obtained valuable information on the interparticle arrangement (nearest neighbors) inside a cluster.

Figure 2A shows example snapshots of a simulation box for both a (+/−) and a (−/−) mixture at ζ = 0.1. For the oppositely charged mixture, a cluster formed around a positively charged microgel (red) by the negatively charged microgels (blue) can be clearly seen, while in the reference case, the similarly charged negative defect (yellow) remains isolated. To quantify the structuring in the system, we consider the radial pair distribution function between the centers of masses of all microgels, *g*(*r*), for the system with increasing ζ. In the oppositely charged mixture at a low ζ = 0.1, whose *g*(*r*) is plotted in Figure 2B (bottom), a peak is observed at *r* ≈ 22σ, where σ is a bead diameter. This distance is much smaller than the diameter of a single model microgel (≈40σ), which indicates that the microgels are significantly overlapping and the peak corresponds to interpenetrating microgels within a cluster. Further peaks correspond to various other distances within one cluster, such as two different matrix microgels interpenetrated with the defect.

**Figure 2:**
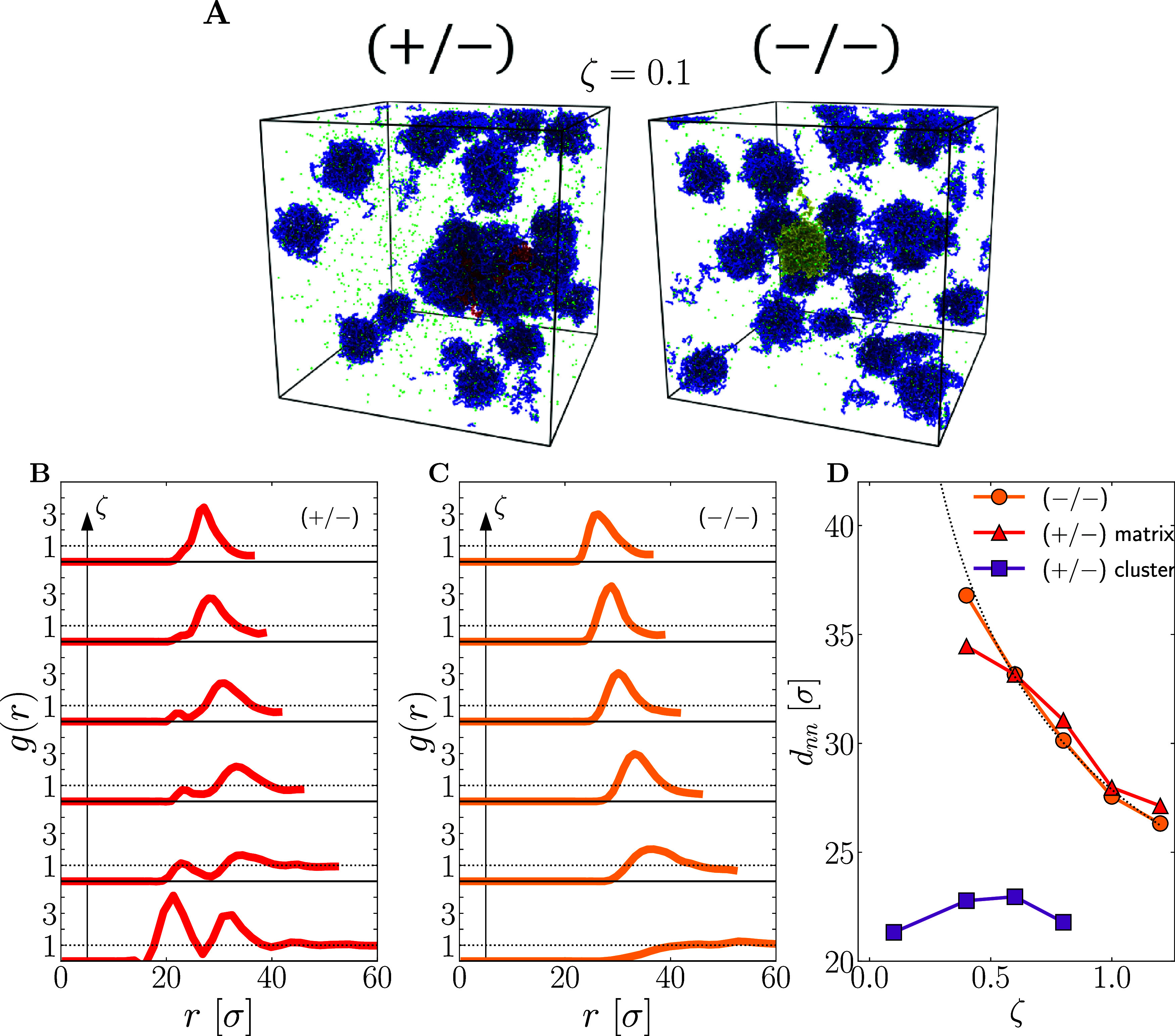
(A) Representative snapshot of both model systems at ζ = 0.1, negatively charged matrix microgels are colored in blue, a positively charged defect microgel in red and a negatively charged defect microgel in yellow. (B) Radial distribution function for various generalized volume fractions (from bottom to top: ζ = 0.1; 0.4; 0.6; 0.8; 1.0; 1.2) of the oppositely charged (+/−) system. (C) Radial distribution function for various generalized volume fractions of the negatively charged (−/−) system. (D) Nearest neighbor distance as a function of generalized volume fraction, obtained from the radial distribution functions. For the oppositely charged (+/−) system, we distinguish between the peaks from the clusters (squares) and from the matrix (triangles). The dotted line indicates the ζ^–1/3^ dependency.

Increasing generalized volume fraction to ζ = 0.4, the cluster peak at small distances decreases in intensity and a second structuring peak at ≈35σ appears. Other cluster peaks are of low magnitude and overshadowed by this emergent peak. For the second peak, it is observed that further compression of the system leads to a systematic increase in intensity accompanied by a shift to smaller distances. The peak is expected to correspond to the structuring of the matrix, since it also arises in a purely negatively charged (−/−) system, as can be seen in Figure 2C, as well as in the literature.^96^

In the binary mixture (+/−), at ζ ≥ 1.0, the two different peaks merge together. Thus, the interparticle distances within a cluster and within the overall matrix become comparable. Here, the (+/−) mixture gradually transitions from a highly heterogeneous system, where dense clusters coexist with a dilute fluid, to a relatively homogeneous system, characterized by similar interparticle distances but different interactions (attractive or repulsive).

Figure 2D shows that the *d*_nn_ values obtained from the second peak of the (+/−) system (red triangles) match those of the (−/−) system (yellow circles) and follow the same *d*_nn_ ∝ ζ^–1/3^ scaling (dashed line). This behavior is consistent with the combination of both electrostatic repulsion between similarly charged species and soft steric repulsion at contact, also seen in experiment. Conversely, the *d*_nn_ values obtained from the first peak of the (+/−) system (purple squares) increase slightly with ζ from ≈22σ to ≈25σ. It appears that the clusters are more attractive at lower microgel concentration and higher free volume in the system.

However, the presence of the low-*q* upturn in all experimental *S*_eff_(*q*) proves that the concentrated system is not strictly homogeneous: the polymer density inside the clusters is higher than in the surrounding medium packed with free microgels, leading to excess scattering from the clusters at low *q* vectors.

In addition, we obtained the flow curves of the H_+_/D_–_ and H_–_/D_–_ mixtures using steady shear rheology. Both systems show shear-thinning behavior with a zero-shear viscosity plateau at low shear rates, η_0_, in the whole investigated ζ-range, Figure S10. This indicates that the suspensions remain fluid-like, and neither a (repulsive) glass transition nor a cluster percolation occurs in them. Furthermore, we observe no additional shear-thinning or viscosity jumps in H_+_/D_–_ mixtures compared to the H_–_/D_–_ mixtures, i.e. no indication of cluster dissociation or aggregation under shear.^97^

### Form Factors of Individual Microgels inside the Clusters

Analysis of the fate of individual microgels inside the assembled clusters was carried out using CV-SANS. Since the samples are prepared in the contrast-matching solvent for D_–_ microgels (80 wt % D_2_O/20 wt % H_2_O), we directly measured the form factors of the protonated microgels, H_+_ or H_–_ in crowded and overcrowded suspensions. Figure 3A shows the SANS intensities, *I*(*q*), for H_+_ microgels in dilute suspension (gray circles) and in H_+_/D_–_ mixtures with increasing ζ (from bottom to top). The fuzzy sphere model, which is typically used to fit form factors of microgels,^68,71,75,83^ can describe the form factor of free H_+_ microgels well (dashed line). However, the model fails in the H_+_/D_–_ mixtures: although a seemingly good quality of the fits can be obtained (see Figure S11A), the fit parameters corresponding to polydispersity and meshsize change in a nonphysical way, as evidenced by Figure S11B,C. This phenomenon may occur in such a large parameter space, where multiple local minima of the χ^2^ function are possible.^98^ In contrast, the fuzzy core–shell model^99^ can describe the form factors of H_+_ microgels in the H_+_/D_–_ mixtures with good accuracy and physically meaningful values of fit parameters, Figure 3A (solid lines) and Table S1. To reduce the number of fit parameters, the meshsize ξ is fixed at 5 nm, which we obtain for H_+_ microgels in dilute suspension. The fit quality is similar or better than the fuzzy sphere model and, importantly, reasonable values of the fit parameters are obtained, Table S1.

**Figure 3:**
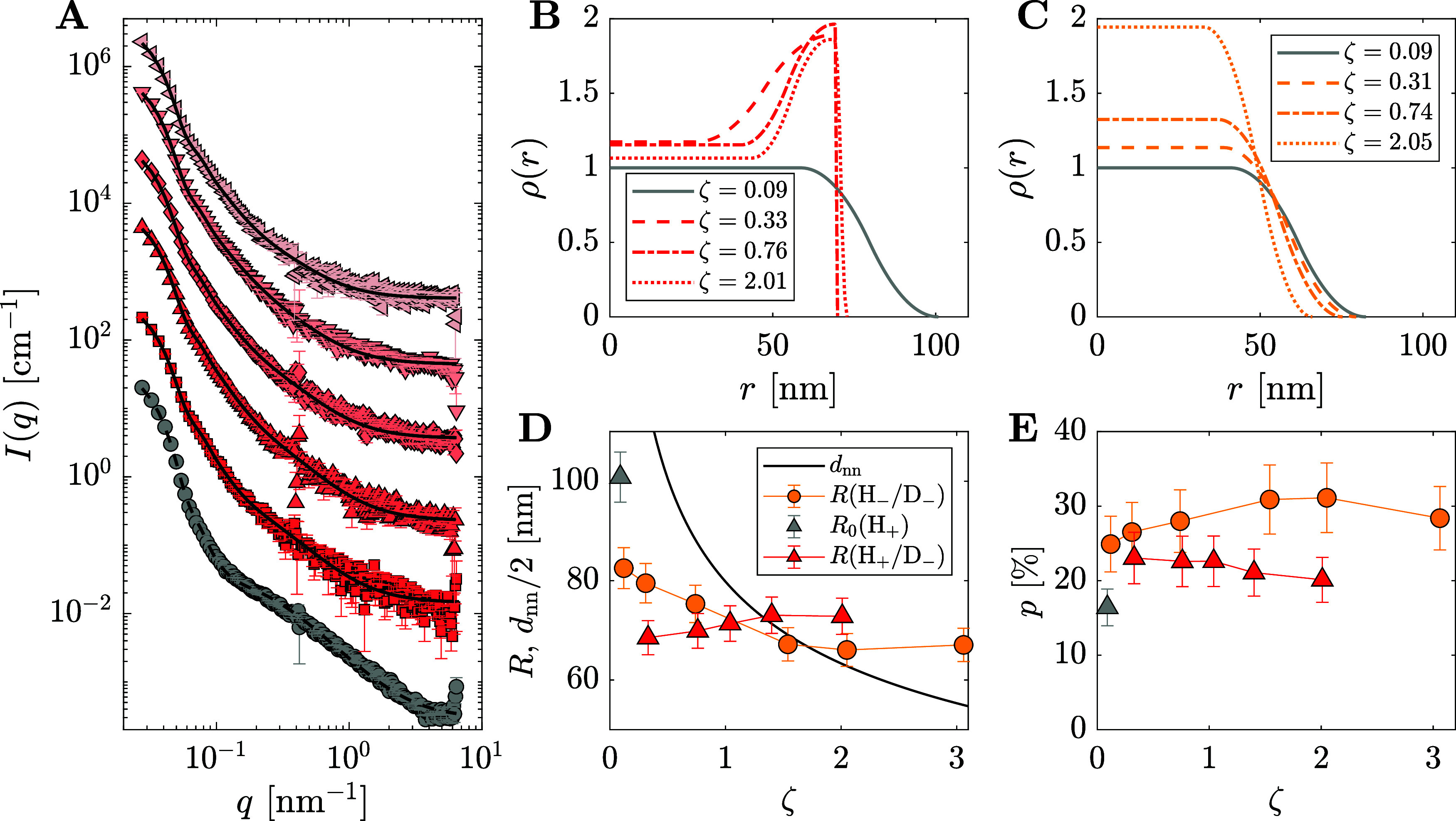
(A) SANS intensities *I*(*q*) vs scattering vector *q* for H_+_ microgels in dilute state (ζ = 0.09 ± 0.02, gray circles) and in H_+_/D_–_ mixtures at ζ = 0.33 ± 0.03 (squares), ζ = 0.76 ± 0.06 (upward triangles), ζ = 1.04 ± 0.09 (diamonds), ζ = 1.40 ± 0.12 (downward triangles) and ζ = 2.01 ± 0.18 (left triangles). Black solid lines are fits with the fuzzy core–shell model. The data are shifted along the *y*-axis for clarity. (B,C) Radial profiles of relative polymer density ρ­(*r*) for H_+_ (B) and H_–_ (C) microgels in dilute state (solid line) and in binary mixtures (dashed, dash-dotted and dotted lines). (D) Radii *R* of H_+_ in dilute state (gray triangle), H_+_ in binary mixtures (red upward triangles) and H_–_ microgels in binary mixtures (yellow circles) vs ζ. Solid line corresponds to the nearest-neighbor distance *d*_nn_. (E) Polydispersity *p* of H_+_ in dilute state (gray triangle), H_+_ in binary mixtures (red upward triangles) and H_–_ microgels in binary mixtures (yellow circles) vs ζ.

The radial density profiles, ρ­(*r*), obtained from the fitting parameters are shown in Figure 3B (red dashed and dotted lines) compared to the ρ­(*r*) in dilute state (gray solid line). In the presence of oppositely charged particles, the H_+_ microgels transform from a fuzzy sphere to a core–shell structure with a dense shell and sharp hard-sphere-like outer boundary. At the same time, the density of the core increases only slightly. This means that strong deswelling of the H_+_ microgels occurs only in their fuzzy shell. In contrast, the H_–_ microgels in concentrated suspensions (H_–_/D_–_) deswell gradually in both the core and the shell and, therefore, retain the fuzzy-sphere structure even in concentrated suspensions, Figure 3C. The corresponding SANS intensities for the H_–_/D_–_ system are shown in Figure S12. The normalized SANS intensities for both mixtures without rescaling along the *y*-axis are shown in Figure S13A,B. Additionally, we show all the data divided point-by-point by the SANS intensity in the dilute case, *I*(*q*,ζ)/*I*(*q*,ζ→0), Figure S13C,D. This representation clearly highlights the differences between the scattering curves at different ζ.

Figure 3D shows the radius of the H_+_ microgels obtained from the fits, R vs ζ, in the dilute state (gray triangle) and in the H_+_/D_–_ mixtures (red triangles). The corresponding size distributions are shown in Figure S14A. First, the radius drops from 101 ± 5 nm in dilute state to 69 ± 3 nm in the mixture at ζ = 0.33 ± 0.03, but then it stays almost constant, slightly increasing to 73 ± 4 nm at ζ = 2.01 ± 0.18. Although microgels can deswell at volume fractions below close packing,^68,69,100^ such a strong decrease in size at such low ζ has not been reported for repulsive microgel suspensions. Indeed, in our similarly charged mixtures-, H_–_/D_–_, we observe a gradual decrease of *R* with increasing ζ, Figure 3D (yellow circles) and Figure S14B. The gradual deswelling in both the core and shell of a microgel is in agreement with the osmotic deswelling mechanism due to the overlap between the counterion clouds that surround the microgels,^101^ reported previously for charged^74,96,100,102,103^ and neutral microgels.^68,69,71,72,86^ In contrast, the peculiar trend of *R* vs ζ in the H_+_/D_–_mixtures must be the direct consequence of the strong electrostatic attraction inside the microgel clusters.

In the literature, a transition from a fuzzy sphere to a core–shell structure with a dense shell has been reported for charged microgels due to complexation with oppositely charged polyelectrolytes^80^ and due to complexation between the core and the shell of the same microgel bearing opposite charges.^81,104,105^ Similarly, computer simulations of polyampholyte Janus-like microgels revealed an increase of polymer density at the contact between the positively and negatively charged networks.^60^ In all these cases, deswelling of the shell was explained by local charge neutralization and entropically favored counterion release. We believe that a similar mechanism is at play in the H_+_/D_–_ clusters: the peripheral polymer chains of the oppositely charged microgels form electrostatic complexes, pushing the particles closer to one another to maximize the contact area and, consequently, the number of released counterions. Thus, the increase of the polymer density in the microgel shell is a result of the two mechanisms: (i) osmotic deswelling due to counterion release and (ii) steric compression due to maximization of contact area between the oppositely charged microgels.

Additionally, we note that the parameter associated with size polydispersity, *p*, is slightly higher in both H_+_/D_–_ and H_–_/D_–_ microgel mixtures compared to the dilute case, Figure 3E. Previous studies have linked an increase of *p* in dense microgel suspensions to formation of facets that result in nonspherical objects.^70,73,74,106^ By contrast, osmotic deswelling typically lowers the apparent size polydispersity.^69,71,72^ Therefore, the compression of microgels inside the clusters might also be accompanied by faceting, and further studies are needed to investigate it, for example using super-resolution confocal microscopy.^95,107^

### Internal Architecture and Counterion Distribution within the Cluster

After verifying that the molecular dynamics simulations of the (+/−) and (−/−) systems lead to comparable results to the one reported by scattering (see Figure 2), we can now use them to extract quantities that are inaccessible experimentally, such as the average polymer density within the defect microgels and within the microgels composing the matrix, as well as the distribution of counterions surrounding the different particles. To mimic the results obtained from SANS fits, radially averaged density profiles centered at the center of mass of the defect microgels are given in Figure 4. For both the (+/−) and the (−/−) system, we show the data at ζ = 0.1 (panels A and B) and ζ = 1.2 (panels C and D), in order to highlight the difference between the low concentration and high concentration regimes. The remaining data can be found in Figures S15 and S16.

**Figure 4:**
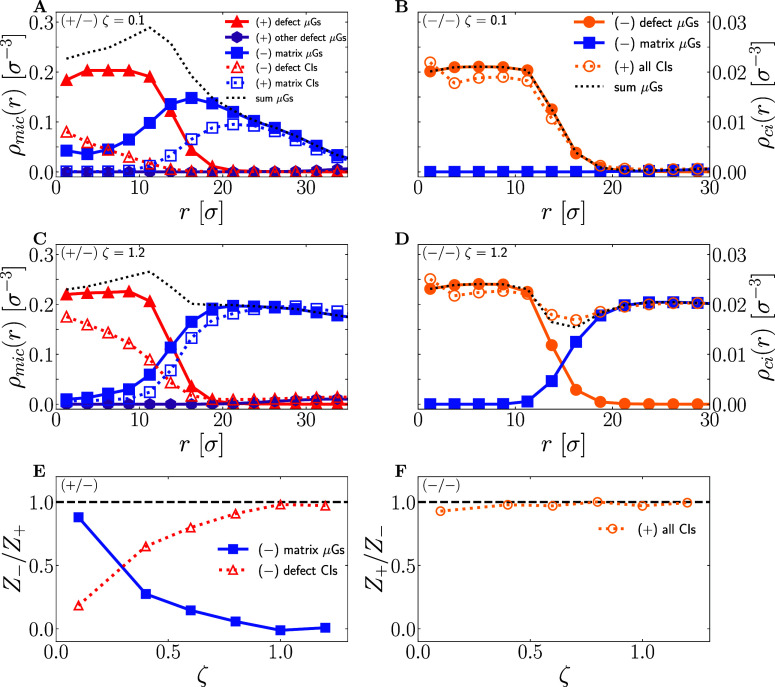
(A) Simulated density profiles of positively charged microgels (μGs) in an oppositely charged matrix at a low generalized volume fraction. Solid red triangles represent the defect and solid blue squares the matrix density, while their sum is given in the dotted black line. Respective counterions (CIs) are given by the corresponding hollow symbols. For visibility the counterion density is scaled by a factor 10, which is also the percentage of charged microgel beads. (B) Simulated density profiles of negatively charged microgels in a similarly charged matrix at a low generalized volume fraction. Solid yellow circles represent the defect and solid blue squares the matrix density, while their sum is given in the dotted black line. (C) Same system as A at a high generalized volume fraction. (D) Same system as B at a high generalized volume fraction. (E) Ratio of negatively charged matrix microgel beads or negatively charged counterions to positively charged defect microgel in a sphere of radius 40 σ around the center of mass of the microgel. The dashed line denotes full charge compensation. (F) Ratio of positively charged counterions to negatively charged defect microgel in a sphere of radius 40 σ around the center of mass of the microgel.

Starting from the oppositely charged (+/−) system at low generalized volume fraction (Figure 4A), the solid red triangles represent the radial density of the positively charged defect microgel and show a typical fuzzy sphere structure. Negatively charged matrix microgels, depicted in solid blue squares, significantly overlap with the central microgel. The two oppositely charged networks strongly interpenetrate to form the cluster. Within the cluster, a strong deficit of counterions, which are depicted in hollow symbols, can be observed. This is the consequence of an entropically favorable counterion release. The charge of the positively charged polymer network can be either compensated with negative counterions or the negative charges of a negatively charged microgel composing the matrix. By interacting with a single microgel that contains a lot of negative charges, many counterions are released to the free volume outside the microgel since this increases the configurational entropy of the system. For the (−/−) reference system at the same ζ in Figure 4B, the central negatively charged microgel depicted in yellow circles does not overlap with the matrix microgel. Furthermore, the radial distribution of counterions follows the density of charged polymer, indicating they are strongly localized.

When increasing the generalized volume fraction, the central defect microgel decreases slightly in radius and has a higher polymer density in the core. In contrast to our experimental observations, this is the case both for a positively and a negatively charged defect (compare Figures S17A,B). As can be seen in Figure 4C, the overlap between defect microgel density (triangles) and matrix microgel density (squares) decreases for high generalized volume fractions. Moreover, the concentration of counterions of the defect (empty triangles) within the cluster becomes larger. Both effects are a consequence of the decrease in free volume in the suspension. After all, the entropy gained from counterion release is larger if more free volume is available to those ions. This also explains why the nearest neighbor distance within a cluster was observed to increase with increasing concentration.

We note that the method of increasing microgel concentration is different in experiments and simulations: in the former, only ζ_D_ is increased, while in the latter, both ζ_H_ and ζ_D_ are increased simultaneously, keeping their ratio constant. Overall, we believe that the attraction within clusters should be stronger at low ζ irrespective of how the latter is varied. The origin of this phenomenon is entropic and related to the amount of free volume available to the released counterions (equal number of positive and negative counterions). Whether this free volume is occupied by both negatively- and positively charged microgels (the case of simulations) or only negatively charged ones (the case of experiments), we expect to have only a minor effect on the amount of entropy gained by the released counterions. Qualitatively, the phenomenon should stay the same.

In the reference (−/−) system, the microgels also interpenetrate each other, as can be seen by the significant overlap of defect and matrix microgel densities (filled circles and squares in Figure 4D). The interpenetration becomes significant for ζ ≥ 0.8, so instead of electrostatic attraction, this interpenetration can be attributed to sterical effects. Microgels get pushed into each other and have to deform in some manner to still fit in the available volume. Consequently, the respective counterion clouds (depicted in hollow circles) also overlap and the overall counterion density becomes almost constant. Thus, any osmotic effects exerted by counterions decrease in strength for high ζ.

These results seem to be in contrast to the results of our SANS experiments, in which we observe a core–shell structure for microgels in an oppositely charged matrix instead of strong interpenetration. To understand this discrepancy, we have to consider the key difference between our simulated model and the real system that is the ratio between the microgel size and its meshsize. Due to computational limitations, one bead–spring microgel consists of 3000 beads and the resulting network is only ≈10 times larger than its meshsize. Especially long dangling chains are able to interpenetrate into a significant portion of the network. For the experimental microgel, this ratio is ≈48, and therefore interpenetration is much more limited.^94^ Consequently, the real microgel is more prone to deform and facet under otherwise similar conditions. Instead of an interpenetration of the network, the outer shells of the real microgels increase in density in order to maximize the contact between oppositely charged chains and, consequently, the counterion release.

As a better type of comparison, we propose the sum of the average defect and matrix densities, which amounts to the total polymer density, shown as the dotted black lines in Figure 4A–D. Here, the fundamental difference between a similarly charged and an oppositely charged microgel system becomes apparent. On the one hand, in a uniformly charged system such as (−/−), there is an effective polymer deficit between microgels. All of their interactions are repulsive and they only come into contact at high concentrations.

In contrast, for the positive defect in negative matrix (+/−), the outer region of the positive defect microgel has the highest polymer concentration in the suspension as shown by the black dashed line for 10 ≲ σ ≲ 20 in Figure 4A,C. Due to their compressibility, the microgels sacrifice internal elastic degrees of freedom to increase the contact area between the oppositely charged chains. Consequently, the polymer density between oppositely charged microgels increases, and more counterions are released into the suspension, which increases the configurational entropy of the system. Although the polymer is spread over the entire suspension at high ζ for both systems, the precise distribution still depends on the charges of the defect microgels. The exact mechanism of deformation depends on the architecture of all microgels in suspension. Our simulated microgels, that are smaller than the one used in experiments, prefer interpenetration, while the experimental results indicate faceting as the dominant response. However, even though these deformations are of a different character, the underlying mechanism to maximize polymer density at the contact between oppositely charged species can be considered the same.

Because the entropy of counterions is the driving force of aggregation, we estimate how the bare charge of the defect network is compensated as a function of volume fraction from the density profiles. All particles within a sphere of radius 40 σ are considered, which ensures that all charges in a microgel, its counterion cloud and the first layer of a potential cluster are included. The resulting quantity is defined as

5 $$Z=\int_{0\sigma }^{40\sigma }\left(\rho \left(r\right)\cdot 4\pi r^{2}\right)dr$$

It has to be considered, that negatively charged microgels in the matrix also bring their own counterions, whose charge has to be subtracted from the matrix microgel charge to get the effective charge. The ratio between the charged microgel beads and the particle types that compensate its charge are computed and depicted in Figure 4E,F.

In the case of only negatively charged microgels, (−/−), the entire charge has to be compensated by counterions, here depicted by hollow circles. At almost all volume fractions the charge is entirely compensated by the counterions in and around the network, only for ζ = 0.1 a small deficit of about 5% is observed. This is consistent with what was reported experimentally in the literature.^69,103^

For the binary system containing positively charged microgels, (+/−), there are instead two different species that can compensate the positive charge: counterions (hollow upside triangles) and charged beads in the matrix microgel networks (solid squares). As already discussed, at lower microgel volume fraction and corresponding higher free volume, network–network interactions are entropically favorable due to the release of counterions. At ζ = 0.3, counterions and charged beads in the matrix microgels contribute almost equally to the compensation of charge and at higher volume fractions counterions are favored as a means of charge compensation. Both kinds of charge-compensation add up to 1.0, so charge neutrality is always achieved. Nonetheless, when the computation is repeated for a reduced sphere radius that includes solely the central microgel, excluding the initial layer of surrounding matrix microgels, only 70% of the charges are compensated. The microgel clusters as a whole achieve charge neutrality, yet they show an uneven distribution of charges.

## Conclusion

In this study, we characterized the structural changes of individual soft polyelectrolyte microgels inside colloidal clusters using DLS, SLS, SAXS, SANS with contrast variation, and molecular dynamics simulations. The clusters were assembled electrostatically from highly asymmetric binary mixtures of a few positively charged protonated microgels (defects) and a majority of negatively charged deuterated microgels. As a reference, mixtures of protonated and deuterated negatively charged microgels were used.

In the oppositely charged mixtures, SAXS data combined with molecular dynamics simulations show a coexistence of irregular clusters having a low fractal dimension (well below the DLCA limit) and a repulsive microgel fluid. By contrast-matching the deuterated microgels in SANS, we directly measured the form factors of the few protonated microgels in concentrated suspensions. SANS results show anomalously strong compression of the microgels within the clusters, which becomes slightly weaker with increasing volume fraction. The compression mainly occurs in the fuzzy shell of the microgels, whereas the highly cross-linked core remains almost unaffected, resulting in a core–shell architecture. In contrast, the reference similarly charged mixtures show osmotic deswelling in both core and shell, in agreement with previous studies.^68,71,84,86,94^

Molecular dynamics simulations reveal that a strong charge compensation occurs between the oppositely charged networks inside a cluster, leading to an entropically favorable counterion release. To maximize the contact between the oppositely charged species, the microgels interpenetrate, resulting in a high polymer density at contact. Simulations show a more pronounced interpenetration than what observed experimentally, where shell compression dominates. This discrepancy is due to the difference in the ratio between the total size and the meshsize of a microgel, which is five times higher for the experimental system compared to the simulated model. However, both the compression of the shell or the interpenetration occur due to the same physical reason: the oppositely charged microgels maximize the number of released counterions, and thus the configurational entropy of the system, through maximizing the contact between the oppositely charged networks.

Our study highlights the fundamental difference in how microgel softness manifests itself in the presence of attractive or repulsive electrostatic interactions. The observed shell compression or interpenetration can influence the valency and shape of colloidal molecules,^39–41^ porosity and strength of colloidal gels^7,29^ or lead to spontaneous appearance of shape anisotropy in supraparticles.^61,62^ The stimuli-responsiveness of such systems may change due to counterion release, in particular the pH, ionic strength and temperature responsiveness. Further studies could focus on quantifying the strength and directionality of interparticle contacts and connecting them to mesoscopic structures and mechanics of the self-assembled systems. Furthermore, the pH-responsiveness of the weak polyelectrolyte microgels can also allow us to create a system capable of forming or breaking up aggregates on demand.

Formation of similar aggregates and clusters is also fundamental for protein and antibody solutions, where an uneven distribution of surface charges (“patchiness”) often drives the aggregation.^64–67^ Therefore, our study can help to understand these more complex systems, in particular how electrostatic complexation can affect the conformation of a single protein. Possibly, further parallels between microgels and antibodies aggregation can be observed at interfaces, where the latter can build strong viscoelastic films with a soft glassy nature.^108,109^ In this context, polyampholyte microgels, where both charged groups are contained within the same network,^60,81,105,110^ will be a better model system for antibodies and a natural extension of microgel clusters.

## Experimental Section

### Materials

*N*-Isopropylacrylamide (NIPAm, 97%, recrystallized from *n*-hexane), methacrylic acid (MAAc, 99%), *N*,*N′*-methylenebis­(acrylamide) (BIS, 99%), and 2,2′-azobis­(2-methylpropionamidine)­dihydrochloride (AMPA, 97%) were purchased from Merck (Sigma-Aldrich). Deuterated *N*-isopropyl-D7 acrylamide (D7-NIPAm, 97%, used as received) was obtained from Polymer Source Inc. (Canada), while *N*-(3-aminopropyl)­methacrylamide hydrochloride (APMH, >98%) was provided by abcr GmbH. 2,2′-Azobis­(*N*-(2-carboxyethyl)-2-methylpropionamidine)­tetrahydrate (ACMA, min. 95%) was obtained from Wako Chemicals. The surfactants cetyltrimethylammonium bromide (CTAB, ≥98%) and sodium dodecyl sulfate (SDS, ≥99%) were purchased from Merck (Sigma-Aldrich). All chemicals except NIPAm were used as received.

### Microgel Synthesis

The microgels were synthesized by free radical precipitation polymerization analogous to previously published protocols.^61,111^ The chemicals used for each microgel species along with the corresponding amounts and synthesis parameters are specified in Table 2. To compensate that in contrast to D7-NIPAm, NIPAm is prone to self-cross-linking,^33^ the deuterated D_–_ microgels were synthesized with a slightly increased BIS cross-linker content (2 mol % in protonated H_+_ and H_–_ microgels vs 3 mol % in deuterated D_–_ microgels). For all microgel species, surfactants were added to the reaction mixtures to obtain microgels of suitably small and comparable size. For the weakly basic, i.e. cationic microgels (H_+_), NIPAm (90.0 mol %), APMH (10.0 mol %), the cross-linker BIS (2.0 mol %), and the surfactant CTAB (35% of CMC) were dissolved in HPLC-grade water before sodium hydroxide solution (NaOH_(aq)_) was added to adjust the pH value to pH ≈ 10. The mixture was heated to 70 °C and purged with nitrogen for 45 min while stirring continuously. The initiator AMPA (1.5 mol %) was dissolved in water and separately purged with nitrogen. The polymerization was initiated by injecting the AMPA solution into the reaction mixture. The reaction was allowed to proceed for 4 h at 70 °C under nitrogen atmosphere and stirring. After cooling overnight, the microgel suspension was first transferred into dialysis tubes (cellulose, MWCO: 12–14 kDa) and dialyzed against deionized water (water exchanged five times) before subjecting it to ultracentrifugation at 60,000 rpm (2 h) and redispersion in double-distilled water (five times). The weakly acidic, i.e. anionic microgels (H_–_ or D_–_), were synthesized by semibatch polymerization to account for the difference in reactivity between NIPAm/D7-NIPAm and MAAc.^61^ After dissolving NIPAm (90.0 mol %, for H_–_) or D7-NIPAm (90.0 mol %, for D_–_), BIS (2.0 mol % for H_–_ or 3.0 mol % for D_–_), and SDS (25% of CMC) in HPLC-grade water, the pH value of the solution was adjusted to pH ≈ 4.5 by adding hydrochloric acid. Then, the mixture was heated to 70 °C and purged with nitrogen for 45 min while stirring continuously. The initiator ACMA (1.5 mol %) was dissolved in water and separately purged with nitrogen, before transferring the solution into the reaction mixture to start polymerization. After 3 min, MAAc (10.0 mol %) was injected into the reaction mixture. The polymerization proceeded for 4 h at 70 °C under nitrogen atmosphere and stirring. After cooling overnight, the negatively charged microgel suspension was purified identically to the positively charged one (see above).

**Table 2:** Solvent Volume and Chemicals (with Masses *m* and Molar Amounts *n*) Used for the Synthesis of the Three Microgel Species H_+_, H_–_, D_–_

microgel	*V*(H_2_O)	NIPAm/D7-NIPAm	MAAc	APMH	BIS	initiator	surfactant
H_+_	200		-			AMPA	CTAB
		*m* = 2.0369 g		*m* = 0.3573 g	*m* = 0.0617 g	*m* = 0.0814 g	*m* = 0.0255 g
		*n* = 18.0 mmol		*n* = 2.00 mmol	*n* = 0.40 mmol	*n* = 0.30 mmol	*n* = 0.07 mmol
H_–_	200			-		ACMA	SDS
		*m* = 2.0369 g	*V* = 0.169 mL		*m* = 0.0617 g	*m* = 0.1243 g	*m* = 0.1153 g
		*n* = 18.0 mmol	*n* = 2.00 mmol		*n* = 0.40 mmol	*n* = 0.30 mmol	*n* = 0.40 mmol
D_–_	90			-		ACMA	SDS
		*m* = 0.9736 g	*V* = 0.0760 mL		*m* = 0.0416 g	*m* = 0.0560 g	*m* = 0.05203 g
		*n* = 8.10 mmol	*n* = 0.90 mmol		*n* = 0.27 mmol	*n* = 0.14 mmol	*n* = 0.18 mmol

### Dynamic and Static Light Scattering

Dynamic light scattering (DLS) was used to determine the hydrodynamic radius of the microgels, *R*_h_. A multiangle ALV instrument (laser wavelength in vacuum λ_0_ = 633 nm) was used to measure the fluctuations of the scattering intensity as a function of the scattering vector *q* = 4π*n*/λ_0_ sin θ/2, where the scattering angle θ was changed between 30° and 110° degrees in 10° steps. The temperature was controlled using a toluene bath to match the refractive index of glass. The measurements were performed at *T* = (20.0 ± 0.1)°C in 10 mM phosphate buffer solution (pH 7.0; refractive index *n*(λ_0_) = 1.33) filtered through a 0.2 μm pore-size syringe filter (poly­(ether sulfone) membrane).

The intensity autocorrelation functions were analyzed using the second order cumulant method.^112^ The obtained *q*-dependent decay rates, Γ­(*q*) = *D*_0_ *q* ^2^, were used to obtain an average diffusion coefficient, *D*_0_, by performing linear fits of the Γ­(*q*) vs *q* ^2^ data. The hydrodynamic radius was calculated from *D*_0_ using the Stokes–Einstein equation *R*_h_ = *k*_B_ *T*/(6πη*D*_0_), where η is the viscosity of water at the temperature *T* and *k*_B_ is the Boltzmann constant.

To perform the static light scattering (SLS) measurements, stock solutions of individual microgels or binary mixtures were diluted approximately 100-fold with 10 mM phosphate buffer (pH 7.0), so that the overall concentration was *c* < 0.01 wt %. An SLS-Systemtechnik GmbH instrument equipped with a red laser (λ_0_ = 640 nm) was used to measure the scattering intensity as a function of *q*. The scattering angles were chosen between 20° and 150° with steps of 2°. The temperature was controlled using a toluene bath set to *T* = (20.0 ± 0.1)°C.

### Electrophoretic Mobility Measurements

Electrophoretic mobility measurements were performed using the Litesizer 500 instrument (Anton Paar). Microgels were dispersed in 10 mM phosphate buffer solution at pH 7.0 ± 0.1 and filtered through a 0.45 μm pore size syringe filter (poly­(ether sulfone) membrane) before transferring into a Omega cuvette (Cat. no. 225288). The experiments were conducted at *T* = (20.0 ± 0.1)°C, a scattering angle of 15° and an effective applied voltage of 200 V. The electrophoretic mobility for each sample was determined using the default Kalliope software and averaged over four separate measurements.

### Fourier-Transform Infrared Spectroscopy

After lyophilization of the microgels, Fourier-transform infrared (FT-IR) spectra of each sample were measured in the dried state using the PerkinElmer Spectrum 3 FT-IR. Spectroscopic data was obtained in the frequency range of 400–4000 cm^–1^ with a resolving power of 1 cm^–1^. The MAAc amount incorporated into the microgels was then approximated by applying the calibration curve known from literature,^61^ which was determined using different mixing ratios between pNIPAm microgels and pure poly­(methacrylic acid).

### Nuclear Magnetic Resonance Spectroscopy

To obtain proton nuclear magnetic resonance (^1^H NMR) spectra of H_+_ microgels, the lyophilized sample was dissolved in D_2_O at a concentration of 0.67 wt %. The suspension was measured in borosilicate tubes using the Bruker AVANCE 400 (Bruker Corp.) at 400 MHz and analyzed with the software MestReNova. The content of APMH comonomer was determined by comparing the signal of the isopropyl group in NIPAm with the signals from the methyl group in APMH, as reported previously.^61^

### Viscosimetry

Viscosities of the microgel suspensions, η, were measured at *T* = 20 °C in 10 mM phosphate buffer (pH 7.0) as a function of weight concentration, *c*, using a 0c Ubbelohde viscometer (SI Analytics). The η vs *c* dependence was fitted with the Einstein–Batchelor equation^113^ to obtain the conversion constant *k* ^69,88^

6 $$\frac{\eta }{\eta_{s}}=1+2.5kc+5.9{\left(kc\right)}^{2}$$

where η_s_ is the solvent viscosity. The generalized volume fraction of the microgels, ζ, is calculated as follows: ζ = *kc* (see also Supporting Information, Section S3).

### Preparation of the Binary Mixtures of Microgels

All microgels were dissolved in a phosphate buffer solution, pH 7.0 and ionic strength of 10 mM, prepared from NaH_2_PO_4_·H_2_O and K_2_HPO_4_.^114^

The binary mixtures of oppositely charged microgels were prepared by slowly mixing two stock solutions using a syringe pump (Cambridge Scientific). Briefly, a concentrated stock solution of deuterated negatively charged microgels (ζ_D,stock_ = 3.92 ± 0.02) was diluted with pure buffer to reach the required value of ζ. The suspension was placed into a small glass vial under vigorous stirring using a magnetic stirring bar. A diluted stock solution of protonated positively charged microgels (ζ_H,stock_ = 0.060 ± 0.006 or 0.12 ± 0.01) was added from a 1 mL Hamilton syringe at a rate of 10 μL/min using the syringe pump. The binary mixtures of similarly charged protonated and deuterated microgels were prepared by mixing two stock solutions with a pipette, because no aggregation can occur in this case.

### Small-Angle Neutron and X-ray Scattering

The small-angle neutron scattering (SANS) measurements were performed using the D22 instrument at the Institut Laue-Langevin (ILL, Grenoble, France). The sample-to-detector distance was set to *d*_SD_ = 17.6 m for the back (^3^He) detector and 1.4 m for the front detector. The neutron wavelength was λ = 0.6 nm, so that the *q*-range was between 2.8 × 10^–2^ and 6.4 nm^–1^. The wavelength resolution, Δλ/λ, is fixed at 10% fwhm and depends on the neutron velocity selector. The total *q*-resolution is given by^115^

7 $$\Delta q=-q\left(\frac{\delta \lambda }{\lambda }\right)+\left(\frac{4\pi }{\lambda }\right)\cos\theta \Delta \theta$$

where 2θ corresponds to the scattering angle and Δθ is the width of the incident neutron beam. For the SANS experiments reported here, Δ*q*/*q* was between 4% at the highest *q* and 29% at the lowest *q*.

The synchrotron small-angle X-ray scattering (SAXS) experiments were performed at the CoSAXS beamline at the 3 GeV ring of the MAX-IV Laboratory (Lund, Sweden).^116^ A *q*-range between 1 × 10^–2^ and 7 × 10^–1^ nm^–1^ was resolved using a photon energy of 12.4 keV and a sample-to-detector distance of 10 m. Data was recorded using an Eiger2 4 M detector with pixel size of 75 μm × 75 μm. A python-based code was used to convert the 2D images to 1D profiles.

The data were fitted with either the fuzzy-sphere model^83^ or the fuzzy core–shell model^99,117,118^ using the customized Matlab-based software FitIt!.^35^ In the fuzzy-sphere model, a particle of radius *R* is composed of a homogeneous core of radius *R*_c_ and a fuzzy shell of width 2σ_out_, *R* = *R*_c_ + 2σ_out_. This profile is obtained by convolving a box-profile with a Gaussian of width 2σ_out_. In the fuzzy core–shell model, two fuzzy spheres with the same origin but different scattering length densities are superimposed. The resulting particle of radius *R* consists of a homogeneous core of radius *R*_c_, an interpenetrating layer between the core and the shell of the length 2σ_in_, a homogeneous shell of length *w*_shell_ and a fuzzy outer surface of the length 2σ_out_. In total, *R* = *R*_c_ + 2σ_in_ + *w*_shell_ + 2σ_out_. The size polydispersity in both models is accounted for by convolving the form factor with a Gaussian distribution of particle size. An additional Lorentzian term with an average meshsize ξ is added to the models to account for density inhomogeneities of the polymer network, which contribute to scattering in the high *q* region. Finally, the models are convolved with a Gaussian function accounting for the instrument resolution.^119^

### Rheology

Rheological measurements were performed on the Kinexus Pro rheometer (Netzsch) using a 40 mm 1° cone–plate geometry. To obtain the flow curves of the samples, shear rate sweeps were performed between 0.5 s^–1^ and 1000 s^–1^. Only the data with torque values τ > 0.8 μNm were used for further evaluation. Prior to each measurement, a shear-rejuvenation protocol was applied to erase the mechanical history of the sample. A constant shear rate of 1000 s^–1^ was applied for 1 min followed by 5 min equilibration at (20.0 ± 0.1)°C. A solvent trap was used to prevent evaporation.

### Molecular Dynamics Simulation

For our molecular dynamics simulations, we use the well-established bead–spring model for polymers to represent a microgel network.^38,120,121^ The networks consist of regular beads of weight 100 g/mol and normalized size σ, each having one or two neighbors, and cross-linker beads, which are connected to four neighboring beads. The networks are generated by randomly placing beads within a spherical confinement. Starting from the cross-linkers, nearby beads are connected while ensuring that the formation of small loops is avoided.^89,122^ Afterward, 10% of the regular beads are randomly selected to be charged, since the synthesis performed leads to a homogeneous distribution of the charged comonomer as well.^82^ An equivalent number of oppositely charged counterions are randomly distributed within the simulation box. These counterions correspond to sodium and chloride ions, whose primary mode of interaction is through their respective electric charges. We additionally set their diameter to 1.0σ, as this parameter has minimal impact on the simulation results and falls within the size range of the ion together with its first hydration shell. Furthermore, since counterion dynamics are not investigated in this study, the counterions are, for simplicity, given the same mass as the monomer beads. All beads interact via the short-ranged repulsive Weeks–Chandler–Andersen potential, which accounts for excluded volume effects

8 $$U_{WCA}\left(r\right)=4\epsilon \left({\left(\frac{\sigma }{r}\right)}^{12}-{\left(\frac{\sigma }{r}\right)}^{6}\right)+\epsilon ,\text{if}\;\frac{r}{\sigma }\leq \sqrt[{6}]{2}$$

Here, *r* is the distance between beads, given in units of the bead diameter σ, and ϵ = 1*k*_B_ *T* serves as the unit of energy, where *k*_B_ and *T* are the Boltzmann’s constant and the absolute temperature, respectively. Beads connected by a bond further interact with a finitely extensible nonlinear elastic (FENE) spring potential (eq 9)^123^

9 $$U_{FENE}\left(r\right)=-kR_{\max}^{2}\cdot \ln\left(1-{\left(\frac{r}{R_{\max}\sigma }\right)}^{2}\right)$$

The spring constant and the maximum bond extension are set to *k* = 15*k*_B_ *T*σ^–2^ and *R*_max_ = 1.5σ, respectively. These parameters have been used successfully to describe the form factor and elastic moduli of microgels in the past.^121,122^ For the computation of electrostatic interactions, the solvent is approximated implicitly as a dielectric continuum, with the dielectric constant set to that of water at 20 °C (ϵ_r_ = 80.1). Since the experiments are performed in buffer, a screened Coulomb potential is applied

10 $$U_{Coulomb}\left(r\right)=\frac{1}{4\pi \epsilon_{0}\epsilon_{r}}\frac{q_{i}q_{j}}{r}\cdot e^{-r/\lambda }$$

Here, *q*_*i*_ and *q*_*j*_ denote the charge of the interacting particles and $\lambda =\sqrt{\frac{\epsilon_{r}\epsilon_{0}k_{B}T}{2N_{A}q_{e}^{2}I}}$ is the Debye length. The ionic strength of the buffer at *I* = 10 mM and a standard monomer size of σ = 0.4 nm correspond to a Debye length of approximately λ ≈ 3.0 nm ≈7.5σ.

All simulations in this work were performed using the software package LAMMPS.^124^

The model system comprises 26 microgel networks, each containing ≈3000 beads, with a 5% cross-link fraction and ≈300 beads with the elemental charge of 1*q*_e_. In the binary (+/−) system, 2 of those microgels carry positive charges while the remaining 24 microgels make up the negatively charged matrix. A Nosé–Hoover thermostat maintains the temperature at *T* = 293.15 K, enforcing an *NVT* ensemble, and time integration is performed with a time step of 2 fs. To efficiently handle the simultaneous computations of long- and short-range interactions, we use an algorithm that incorporates multiple neighbor lists.^125^ The system undergoes equilibration over a duration of 10^6^ time steps, after which all quantities are computed as averages over an additional 2 × 10^6^ time steps.

## Supplementary Material



## Data Availability

All experimental and simulation data used in this manuscript, including LAMMPS input scripts, are available from the RADAR4Chem repository at DOI: 10.22000/c473c3dch9pw45r3. The SANS data are available at DOI: 10.5291/ILL-DATA.9-11-2133.

## Notes

FT-IR and ^1^H NMR spectra; form factors of microgels in dilute suspension (SANS or SAXS); viscosimetry data and fits; reduced SAXS intensities, *I*(*q*), for the concentrated mixtures; average hydrodynamic radii of diluted clusters (DLS); steady-state flow curves incl. a plot of zero-shear viscosity vs ζ; table with fit parameters for SANS data; plots of the H_+_/D_–_ SANS intensity fitted with the fuzzy sphere model; plots of the H_+_/D_–_ SANS intensity; simulated density profiles of the microgels and counterions in (−/−) and (+/−) mixtures; simulated density profiles of total polymer density in (−/−) and (+/−) mixtures (PDF)
