# Supplementary material for: Compression and Interpenetration of Ionic Microgels in Electrostatically Self-Assembled Clusters
Source: ACS Nano. 2026 Jul 24;20(31):21599–614. doi: 10.1021/acsnano.6c00724 (PMC13472330; doi:10.1021/acsnano.6c00724)
Supplement: Supplementary file 1 [file nn6c00724_si_001.pdf]

Supporting Information. Compression and interpenetration of ionic microgels in electrostatically self-assembled clusters

Alexander V. Petrunin,[*,†,‡] Hannah F. Mathews,[¶,§] Tom Höfken,[‡] Philipp Leclercq,[‡] Ralf Schweins,[‖] Pablo Mota-Santiago,[⊥,#] Stefanie Schneider,[‡] Andrij Pich,[¶,§,@] and Andrea Scotti[*,△]

†*Institute of Experimental Physics of Condensed Matter, Heinrich-Heine University, Universitätsstr. 1, 40225 Düsseldorf, Germany*
‡*Institute of Physical Chemistry, RWTH Aachen University, Landoltweg 2, 52074 Aachen, Germany*
¶*DWI - Leibniz Institute for Interactive Materials e. V., Forckenbeckstr. 50, 52074 Aachen, Germany*
§*Institute of Technical and Macromolecular Chemistry, RWTH Aachen University, Worringerweg 2, 52074 Aachen, Germany*
‖*Institut Laue-Langevin ILL, DS/LSS, 71 Avenue des Martyrs, F-38000 Grenoble, France*
⊥*Australian Synchrotron, ANSTO, Clayton Victoria 3168, Australia*
#*MAX IV Laboratory, Lund University, 22100 Lund, Sweden*
@*Aachen Maastricht Institute for Biobased Materials (AMIBM), Brightlands Chemelot Campus, Maastricht University, 6167 RD Geleen, The Netherlands*
△*Division of Physical Chemistry, Lund University, 22100 Lund, Sweden*

E-mail: alexander.petrunin@hhu.de; andrea.scotti@chem.lu.se

S1. Comonomer content

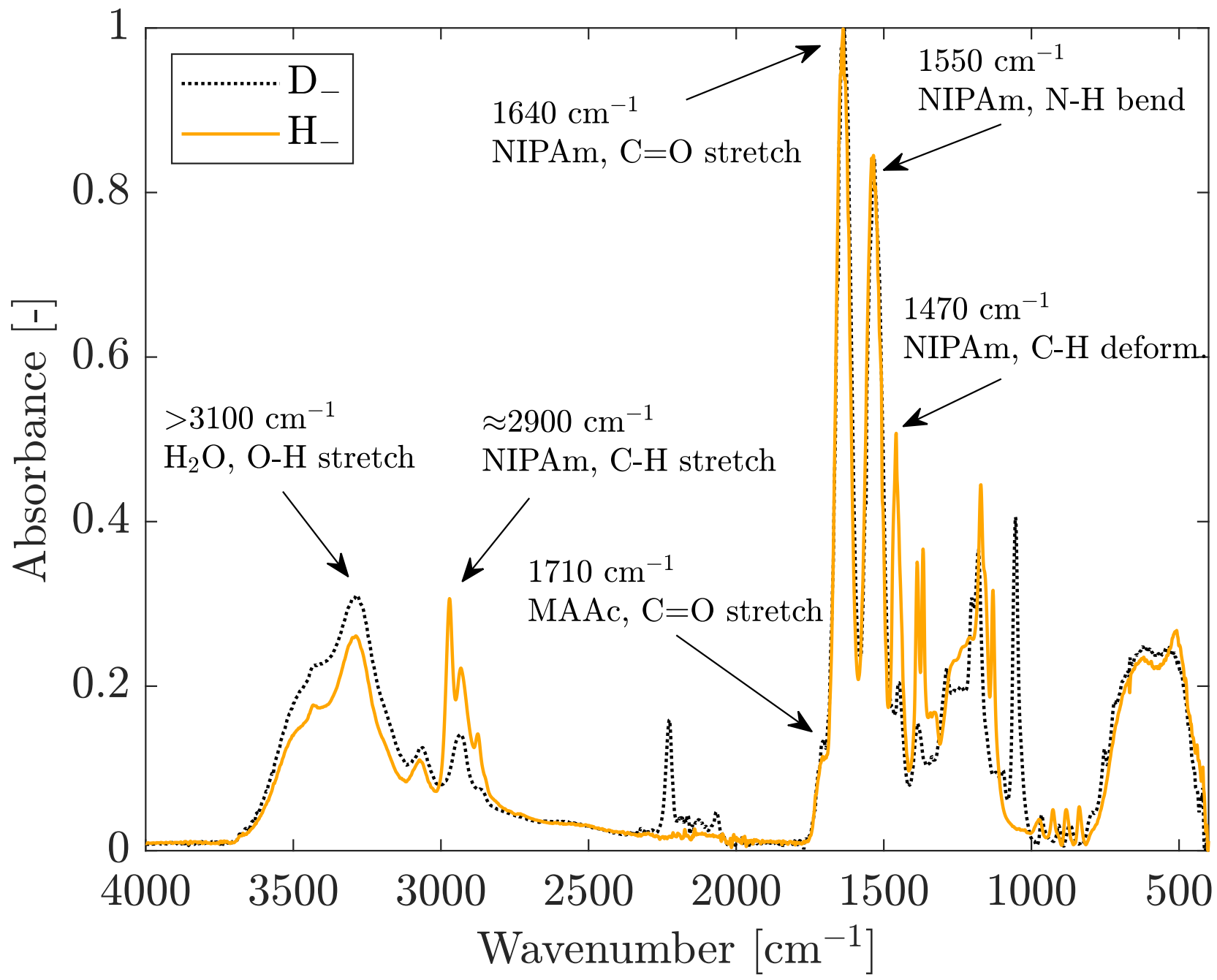

Figure S1: Fourier transform Infrared Spectra of the D− microgels (black dashed line) and H− microgels (yellow solid line) used for the determination of MAAc content.

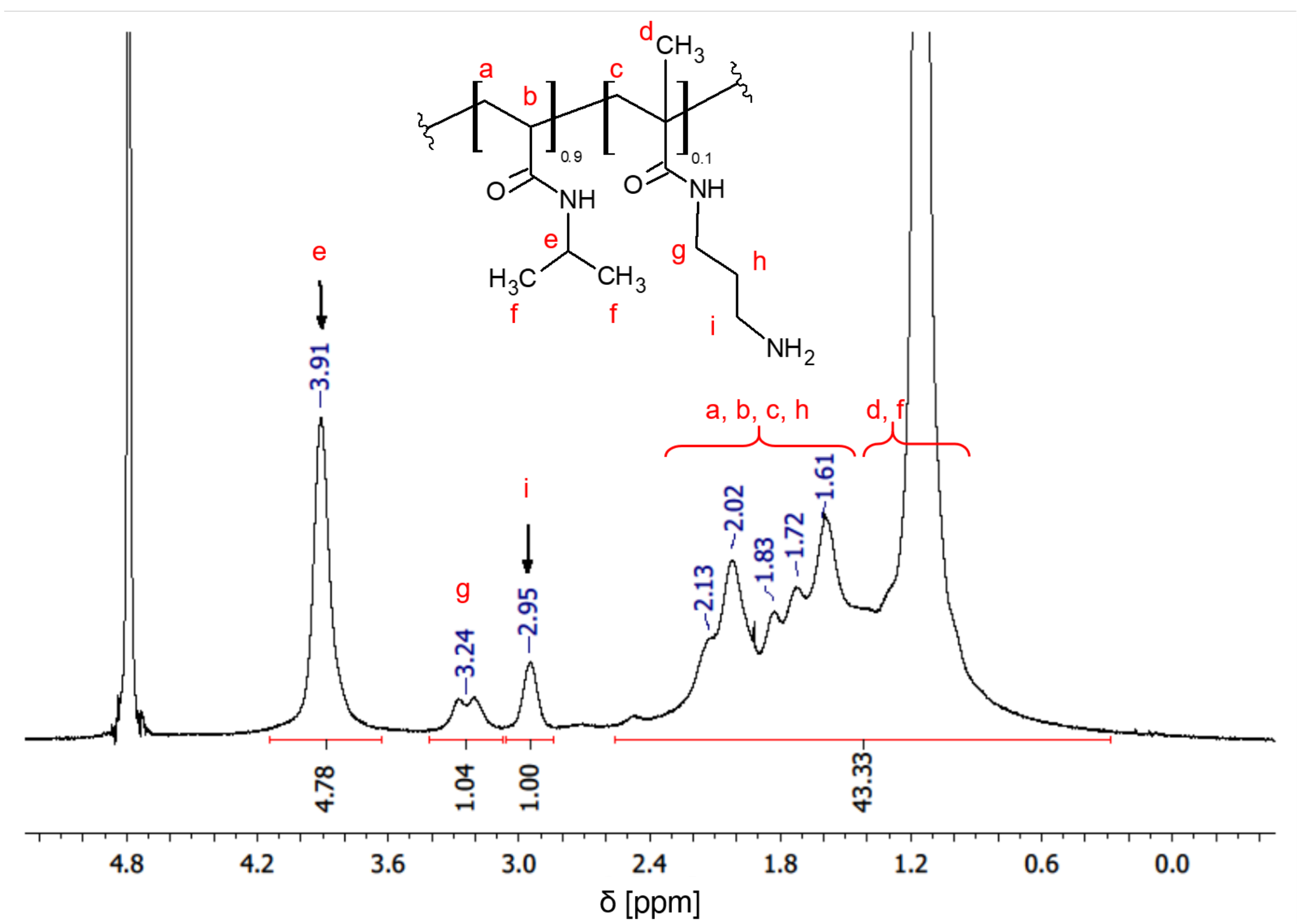

Figure S2: ^{1}H-NMR spectrum of the H_+ microgels in D_2O.

S2. Form factors in dilute suspension

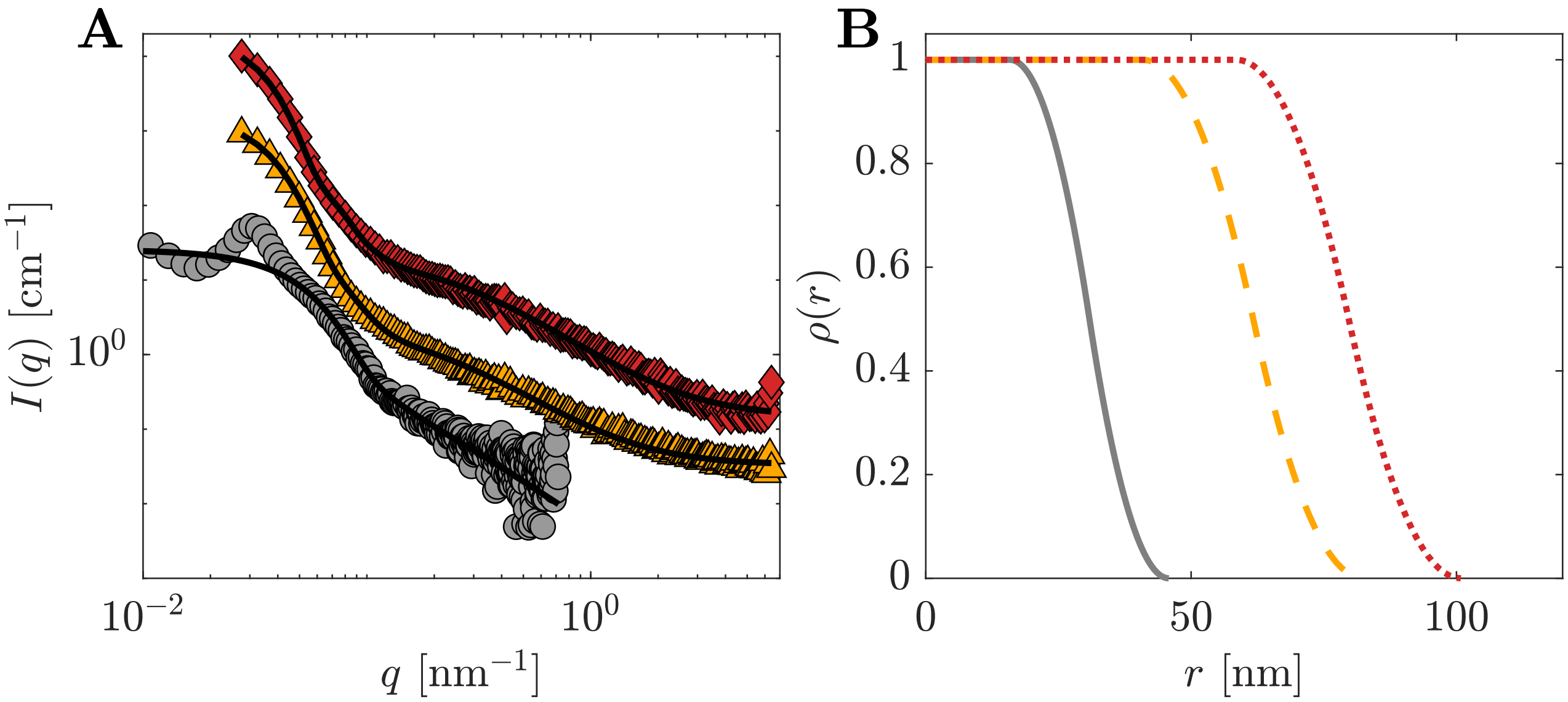

Figure S3: (**A**) Small-angle neutron or X-ray scattering intensities $I(q)$ as a function of the scattering vector q for suspensions of individual microgels: D_- microgels (gray circles, $\zeta = 0.42 \pm 0.04$), H_- microgels (yellow triangles, $\zeta = 0.12 \pm 0.03$), H_+ microgels, (red diamonds, $\zeta = 0.09 \pm 0.02$). The curves are shifter along the y-axis for clarity. (**B**) Radial density profiles $\rho(r)$ of the microgels in dilute condition obtained from the fits: D_- microgels (gray solid line), H_- microgels (yellow dashed line), H_+ microgels (red dotted line).

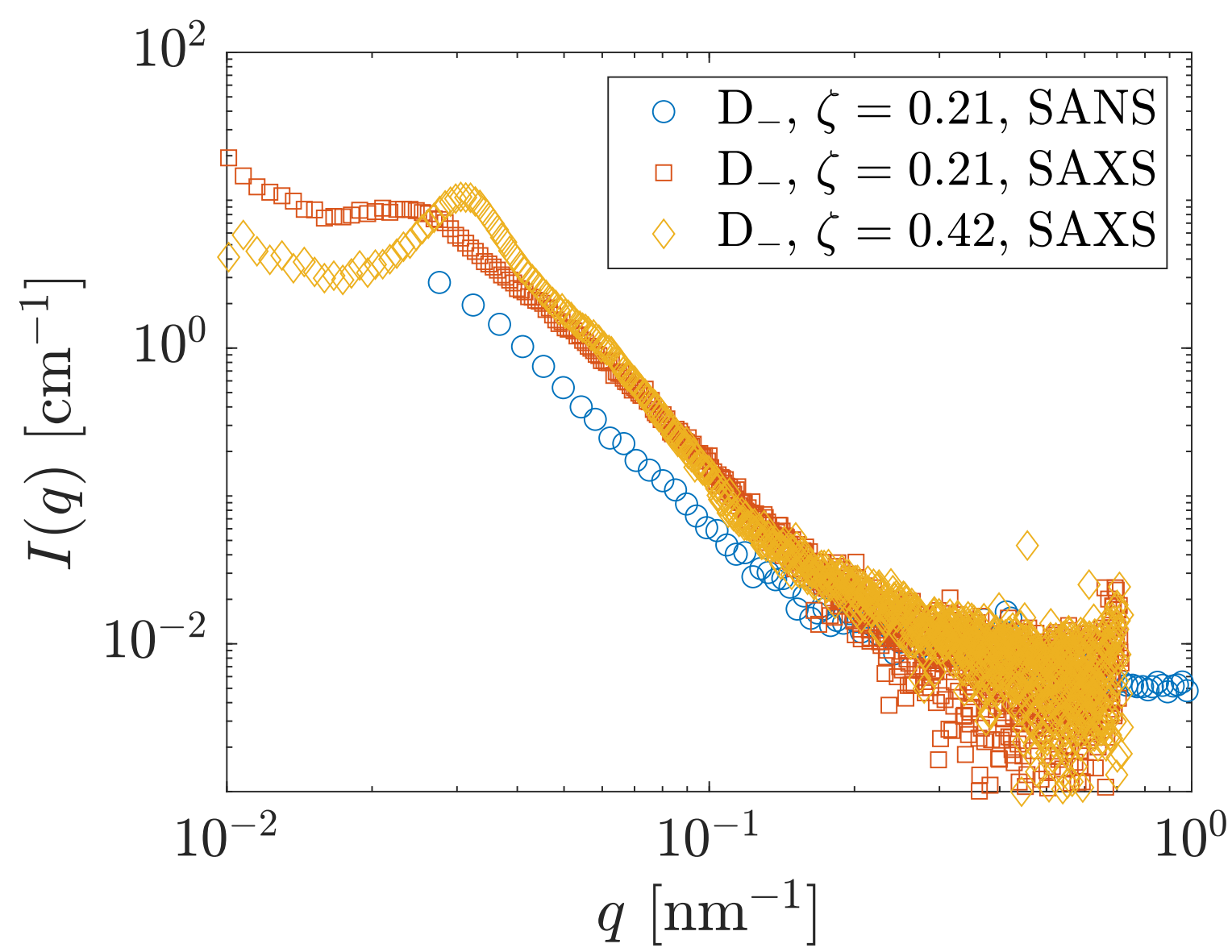

Figure S4: Small-angle neutron and X-ray scattering intensity $I(q)$ *vs.* scattering vector q for D_- microgels at different ζ, as indicated in the legend.

S3. Viscosimetry and Molecular Weight

The volume fraction of microgels is commonly expressed as the generalized volume fraction ζ, which is proportional to weight fraction c, $\zeta = kc$,[1,2] where k is the conversion constant. ζ describes the volume fraction that microgels would occupy in their fully swollen state:

$$\zeta = \frac{Nv_0}{V_{\text{total}}}, \tag{S1}$$

where v_0 is the volume of a fully-swollen microgel, N is the number of microgels in suspension and V_{total} is the suspension volume.

The conversion constant k needed to calculate ζ from weight concentration was determined from the dynamic viscosity of microgel suspensions at low concentrations following the Einstein-Batchelor equation:[3]

$$\frac{\eta}{\eta_s} = 1 + 2.5\zeta + 5.9\zeta^2 = 1 + 2.5kc + 5.9(kc)^2 \tag{S2}$$

where η_s is the solvent viscosity.

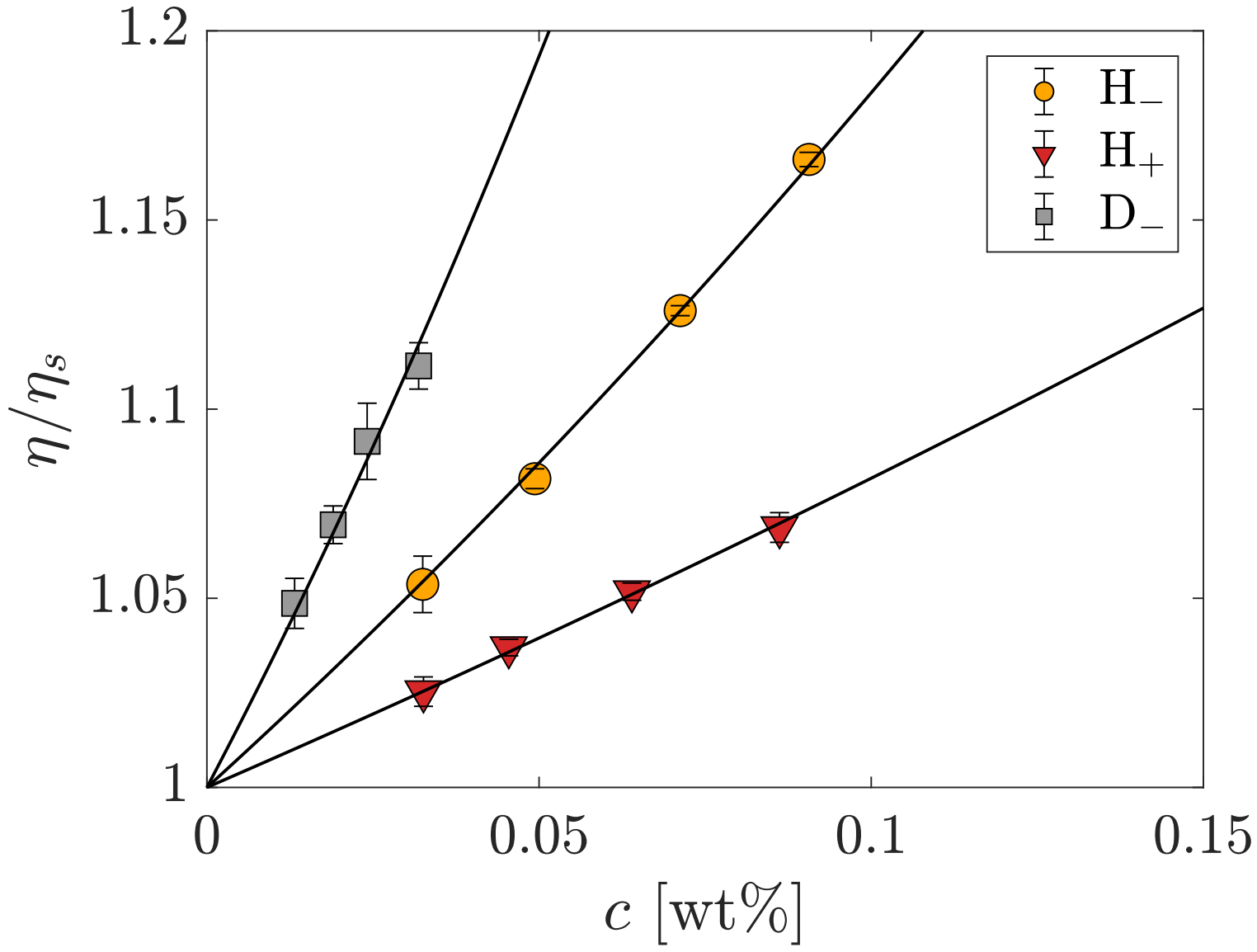

Figure S5: Relative viscosities of the microgel suspensions $\eta_r = \eta/\eta_s$ as a function of the weight fraction in solution c for D_- microgels (gray squares), H_- microgels (yellow circles) and H_+ microgels (red triangles). Solid lines are fits according to the Equation S2.

From viscosimetry and DLS measurements, the molecular weights of the microgels can be estimated as follows:[2,4]

$$M_w = N_A \frac{\rho_s v_{sw}}{k} \tag{S3}$$

where N_A is the Avogadro's number, ρ_s is the density of the solvent, v_{sw} is the volume of a swollen microgel that is obtained using the hydrodynamic radius measured directly by DLS at 20 °C ($v_{sw} = \frac{4}{3}\pi R_h^3$), and k is the viscosimetry conversion constant.

Using the values given in Table 1 of the main manuscript, we obtain $M_w(H_-) = (5.6 \pm 0.3) \cdot 10^7$ g/mol $\rightarrow$ degree of polymerization $N_p(H_-) = (5.0 \pm 0.3) \cdot 10^5$; $M_w(H_+) = (1.3 \pm 0.1) \cdot 10^8$ g/mol $\rightarrow$ $N_p(H_+) = (1.2 \pm 0.1) \cdot 10^6$; $M_w(D_-) = (3.0 \pm 0.4) \cdot 10^7$ g/mol $\rightarrow$ $N_p(D_-) = (2.5 \pm 0.3) \cdot 10^5$. Using the

contents of charged groups determined experimentally, we can estimate the total net charge of our microgels (in experiments): $Q(\mathrm{H}_-) \approx -4.3 \cdot 10^4 \cdot q_e$; $Q(\mathrm{H}_+) \approx 1.1 \cdot 10^5 \cdot q_e$; $Q(\mathrm{D}_-) \approx -2.7 \cdot 10^4 \cdot q_e$.

S4. Characterization of microgel clusters

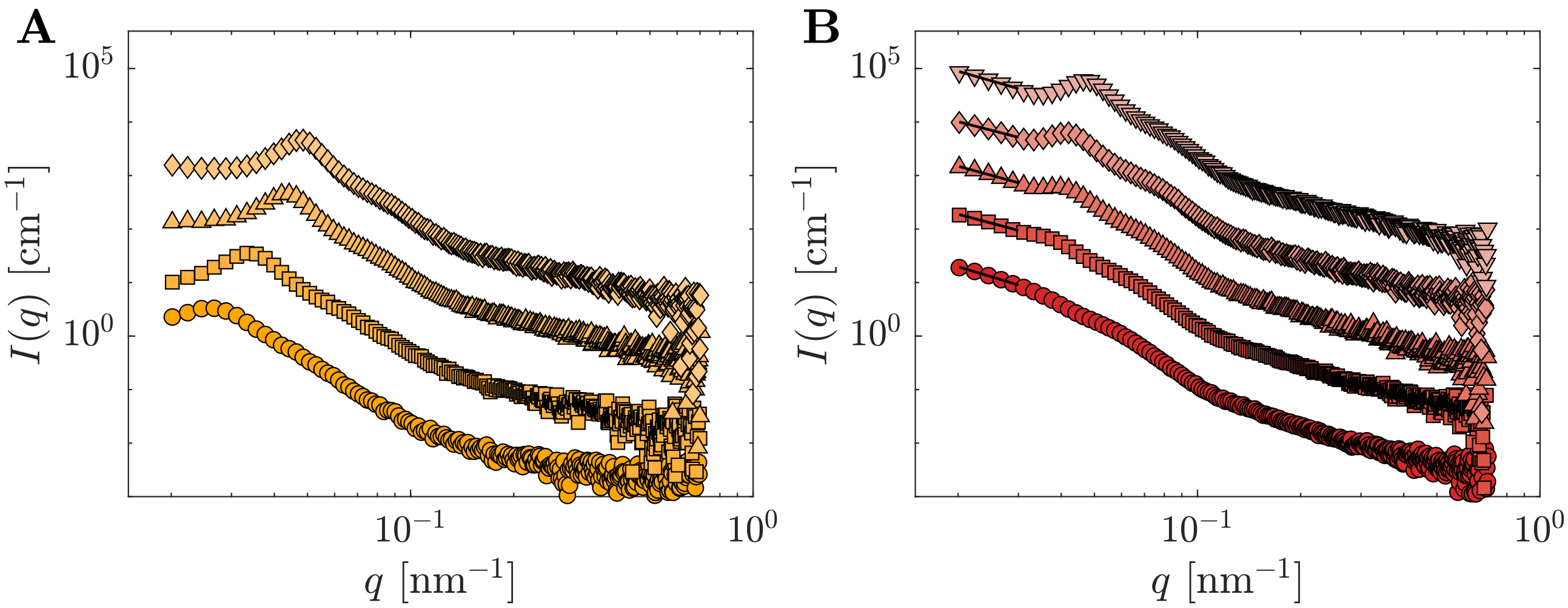

Figure S6: (**A**) SAXS intensities $I(q)$ *vs* scattering vector q for $\mathrm{H}_-/\mathrm{D}_-$ mixtures at $\zeta = 0.31 \pm 0.03$, $\zeta = 0.74 \pm 0.06$, $\zeta = 1.5 \pm 0.1$, $\zeta = 2.1 \pm 0.2$ (from bottom to top). (**B**) SAXS intensities $I(q)$ *vs* scattering vector q for $\mathrm{H}_+/\mathrm{D}_-$ mixtures at $\zeta = 0.76 \pm 0.07$, $\zeta = 1.04 \pm 0.09$, $\zeta = 1.4 \pm 0.1$, $\zeta = 2.0 \pm 0.2$ (from bottom to top). The data are shifted along the y-axis for clarity. Black solid lines correspond to fits using Equation 7.

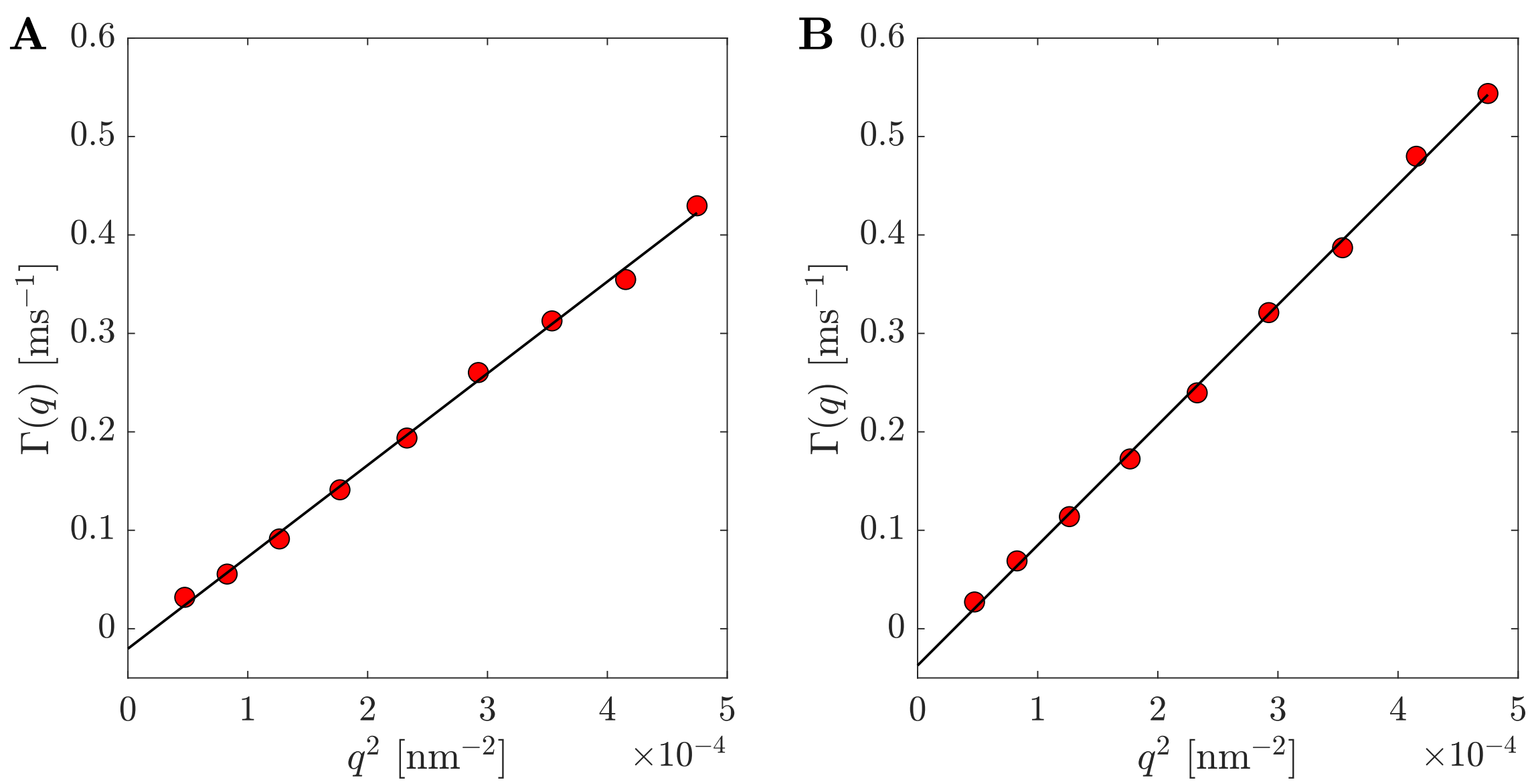

Figure S7: Examples of decay rates obtained from the DLS intermediate scattering functions using the second order cumulant method, $\Gamma(q)$, *vs* the square of the scattering vector, q^2, for diluted $\mathrm{H}_+/\mathrm{D}_-$ samples initially prepared at $\zeta = 0.33 \pm 0.03$ (A) and $\zeta = 2.01 \pm 0.04$ (B). Black lines correspond to linear fits.

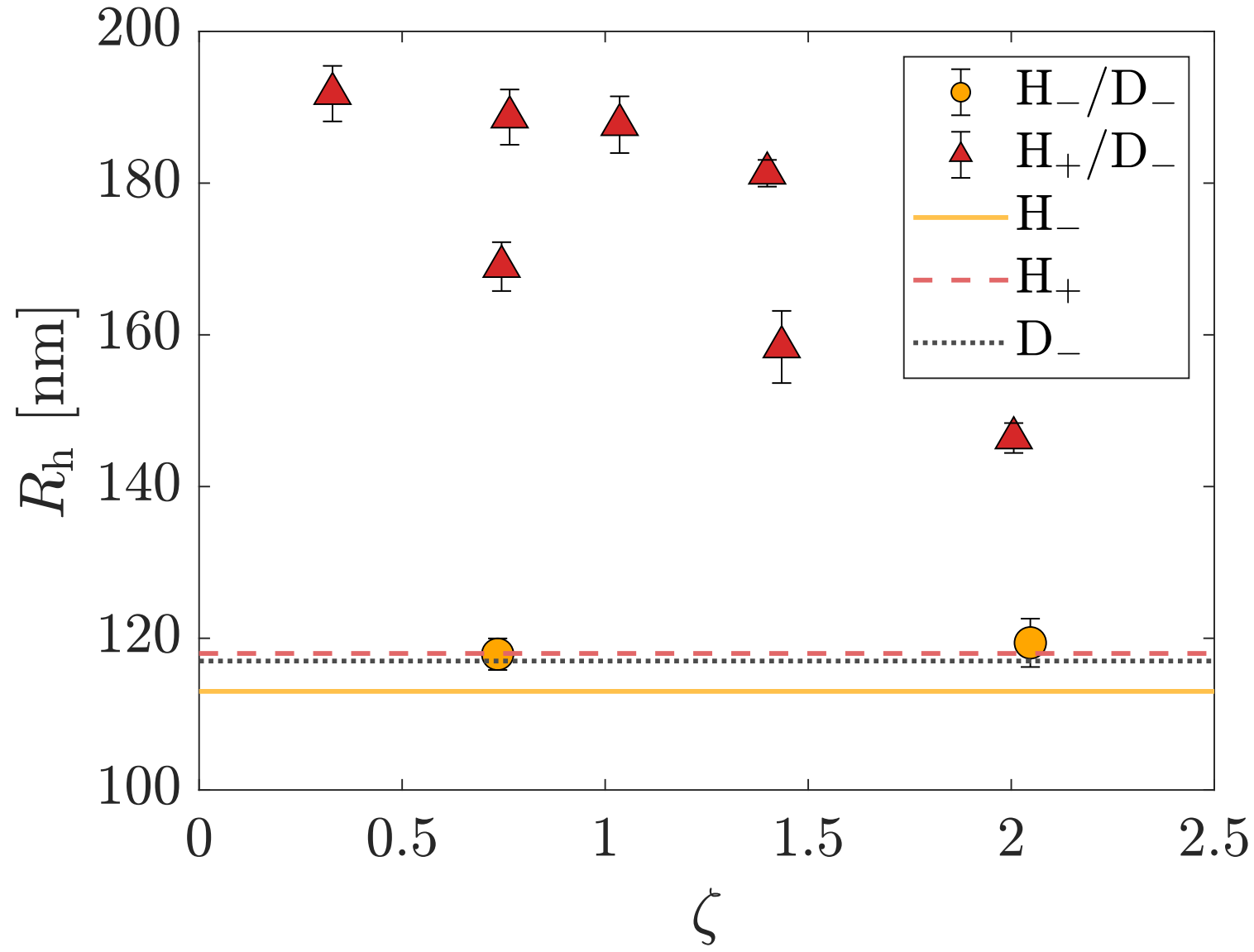

Figure S8: Average hydrodynamic radii R_h of diluted H_+/D_- mixtures (red triangles) and H_-/D_- mixtures (yellow circles) as a function of ζ before dilution. Horizontal lines correspond to R_h of individual microgels: H_- (solid line), H_+ (dashed line) and D_- (dotted line).

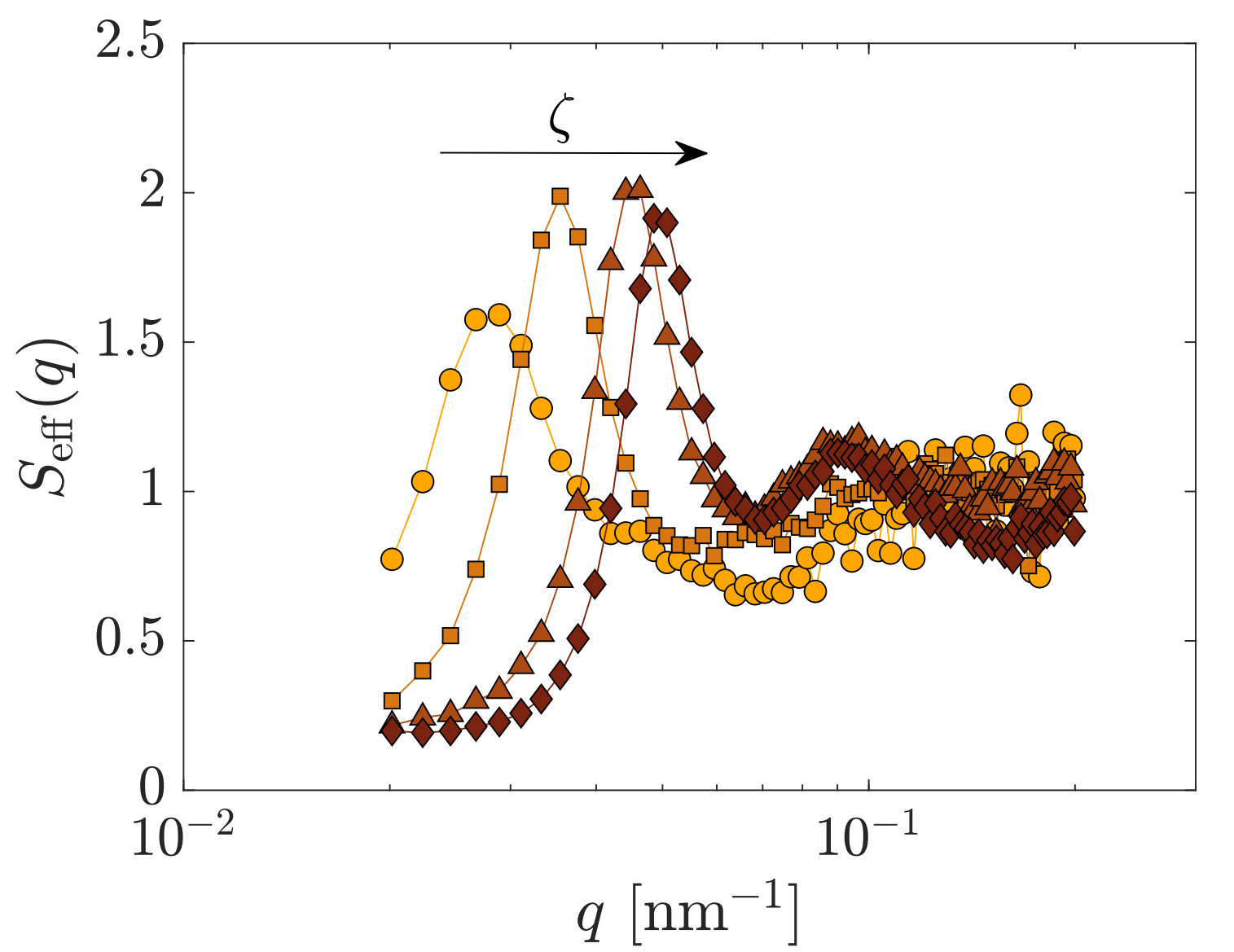

Figure S9: Effective structure factors from SAXS $S_{eff}(q)$ *vs.* scattering vector q for H_-/D_- mixtures at $\zeta = 0.31 \pm 0.03$ (circles), $\zeta = 0.74 \pm 0.06$ (squares), $\zeta = 1.5 \pm 0.1$ (triangles), $\zeta = 2.1 \pm 0.2$ (diamonds).

S5. Rheology

The flow curves of the samples (viscosity η as function of shear rate $\dot{\gamma}$) were fitted with the Cross equation:[5]

$$\frac{\eta - \eta_\infty}{\eta_0 - \eta_\infty} = \frac{1}{1 + (\dot{\gamma}/\dot{\gamma}_c)^m}, \tag{S4}$$

where η_0 is the zero-shear viscosity, η_∞ is the high-shear viscosity, $\dot{\gamma}_c$ is the critical shear rate halfway between η_0 and η_∞ and m is the shear-thinning exponent.

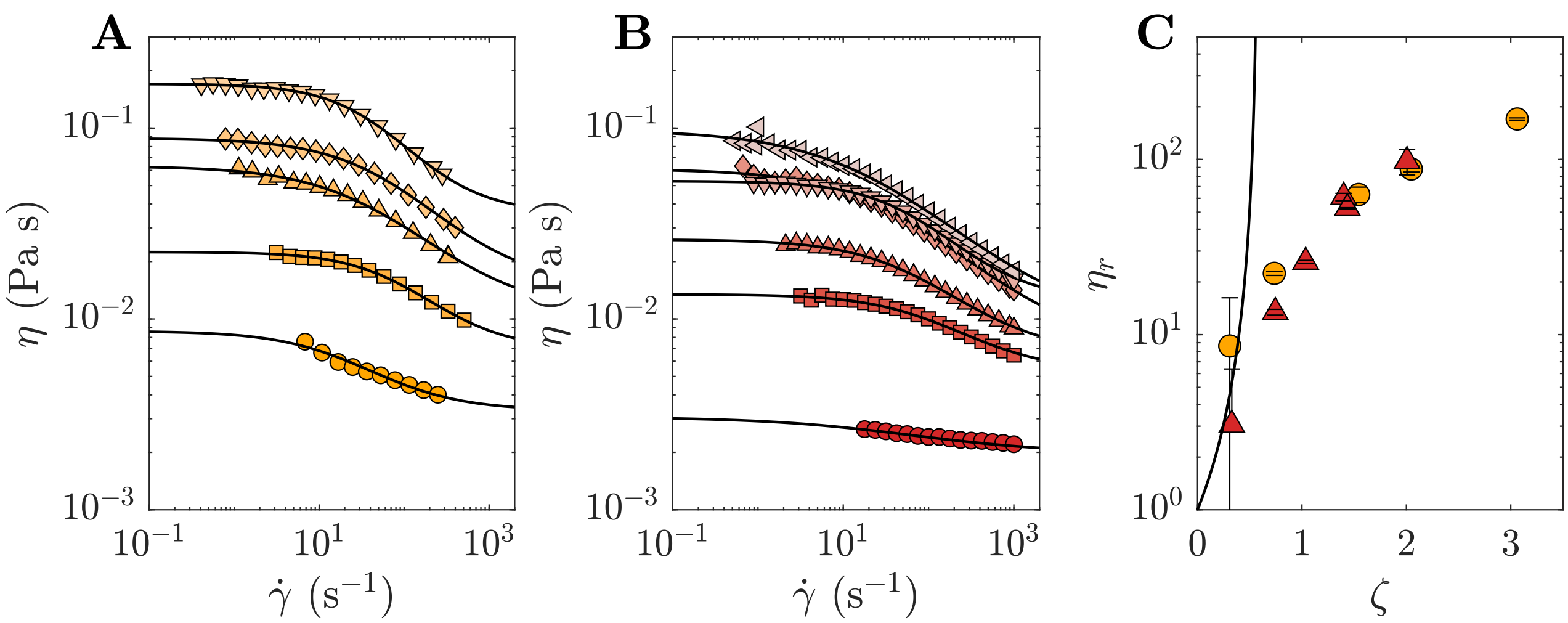

Figure S10: (**A**, **B**) Flow curves of the binary mixtures of protonated and deuterated microgels, H_-/D_- (**A**) and H_+/D_- (**B**). ζ increases from bottom to top. Black solid lines correspond to fits using Equation S4. (**C**) Zero-shear viscosities relative to the solvent $\eta_r = \eta_0/\eta_{solv}$ as a function of ζ for the H_-/D_- mixtures (yellow circles) and H_+/D_- mixtures (red triangles). Black solid line corresponds to the viscosity of hard spheres according to the Quemada model ($\zeta_0 = 0.58$).[6]

S6. SANS in concentrated binary mixtures

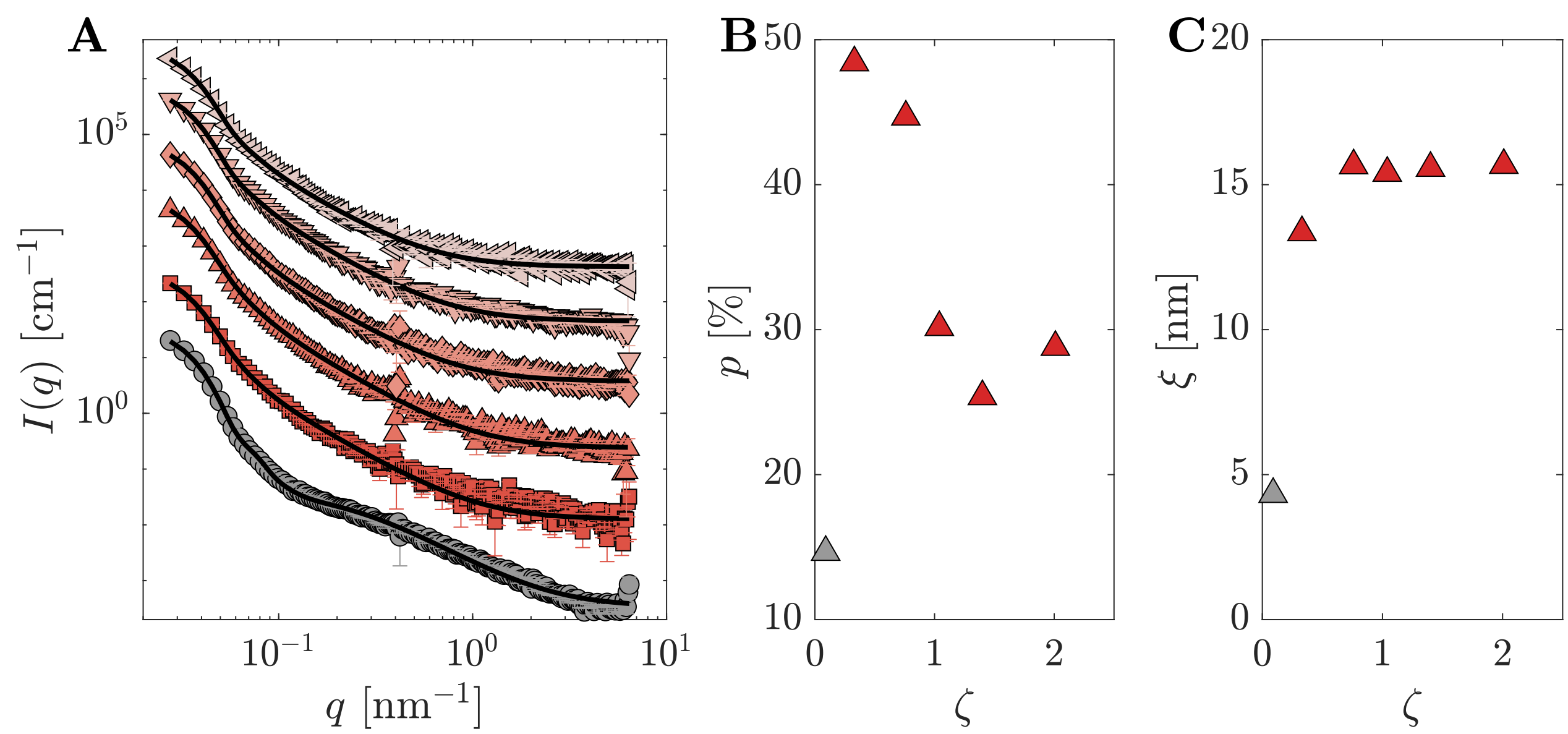

Figure S11: (**A**) SANS intensities $I(q)$ *vs.* scattering vector q for H_+ microgels in dilute state ($\zeta = 0.09 \pm 0.02$, gray circles) and in H_+/D_- mixtures at $\zeta = 0.33 \pm 0.03$ (squares), $\zeta = 0.76 \pm 0.06$ (upward triangles), $\zeta = 1.04 \pm 0.09$ (diamonds), $\zeta = 1.40 \pm 0.12$ (downward triangles) and $\zeta = 2.01 \pm 0.18$ (left triangles). Black solid lines are fits with the fuzzy sphere model. The data are shifted along the y-axis for clarity.(**B**) Polydispersity p of H_+ in dilute state (gray triangle) and H_+ in binary mixtures (red triangles) in binary mixtures (yellow circles) *vs.* ζ according to the fuzzy-sphere model fits. (**C**) Meshsize ξ of H_+ in dilute state (gray triangle) and H_+ in binary mixtures (red triangles) in binary mixtures (yellow circles) *vs.* ζ according to the fuzzy-sphere model fits.

Table S1: Parameters of the SANS form factor fits for concentrated binary mixtures. An asterisk (*) indicates that the parameter was fixed during fitting.

Microgel	ζ	R [nm]	R_c [nm]	$2\sigma_{in}$ [nm]	w_{shell} [nm]	$2\sigma_{out}$ [nm]	ξ [nm]	p [%]
H_-	0.12 ± 0.03	83 ± 4	41 ± 2	–	–	42 ± 2	5 ± 1	25 ± 4
H_-/D_-	0.31 ± 0.03	79 ± 4	41 ± 2	–	–	38 ± 2	5 ± 1	27 ± 4
H_-/D_-	0.74 ± 0.06	75 ± 4	38 ± 2	–	–	37 ± 2	5 ± 1	28 ± 4
H_-/D_-	1.54 ± 0.13	67 ± 3	32 ± 2	–	–	35 ± 2	5 ± 1	31 ± 5
H_-/D_-	2.05 ± 0.18	66 ± 3	33 ± 2	–	–	33 ± 2	4 ± 1	31 ± 5
H_-/D_-	3.06 ± 0.27	67 ± 3	38 ± 2	–	–	29 ± 1	5 ± 1	28 ± 4
H_+	0.09 ± 0.02	101 ± 5	42 ± 2	–	–	59 ± 3	4 ± 1	16 ± 2
H_+/D_-	0.33 ± 0.03	69 ± 3	26 ± 1	42 ± 2	0 ± 1	0 ± 1	5^*	23 ± 3
H_+/D_-	0.76 ± 0.06	70 ± 4	40 ± 2	29 ± 1	1 ± 1	0 ± 1	5^*	23 ± 3
H_+/D_-	1.04 ± 0.09	71 ± 4	43 ± 2	27 ± 1	1 ± 1	0 ± 1	5^*	23 ± 3
H_+/D_-	1.40 ± 0.12	73 ± 4	50 ± 3	18 ± 1	1 ± 1	4 ± 1	5^*	21 ± 3
H_+/D_-	2.01 ± 0.18	73 ± 4	44 ± 2	24 ± 1	1 ± 1	4 ± 1	5^*	20 ± 3

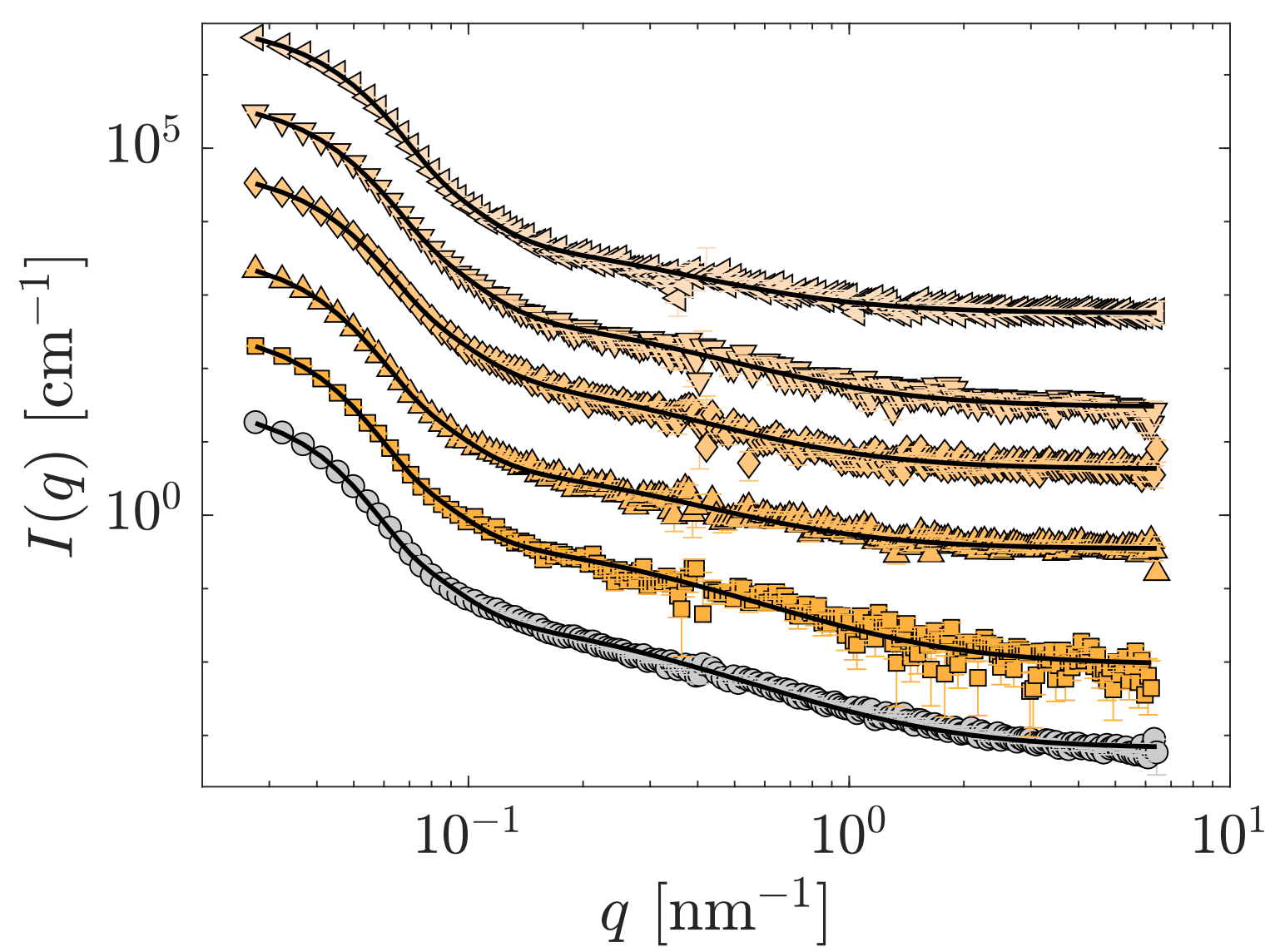

Figure S12: SANS intensities $I(q)$ *vs.* scattering vector q for the H_- microgels at $\zeta = 0.12 \pm 0.03$ (gray circles) and H_-/D_- mixtures at $\zeta = 0.31 \pm 0.03$, 0.74 ± 0.06, 1.54 ± 0.13, 2.05 ± 0.18, 3.06 ± 0.27 (from bottom to top). Black solid lines correspond to fits with the fuzzy sphere form factor. The curves are shifted along the y-axis for clarity.

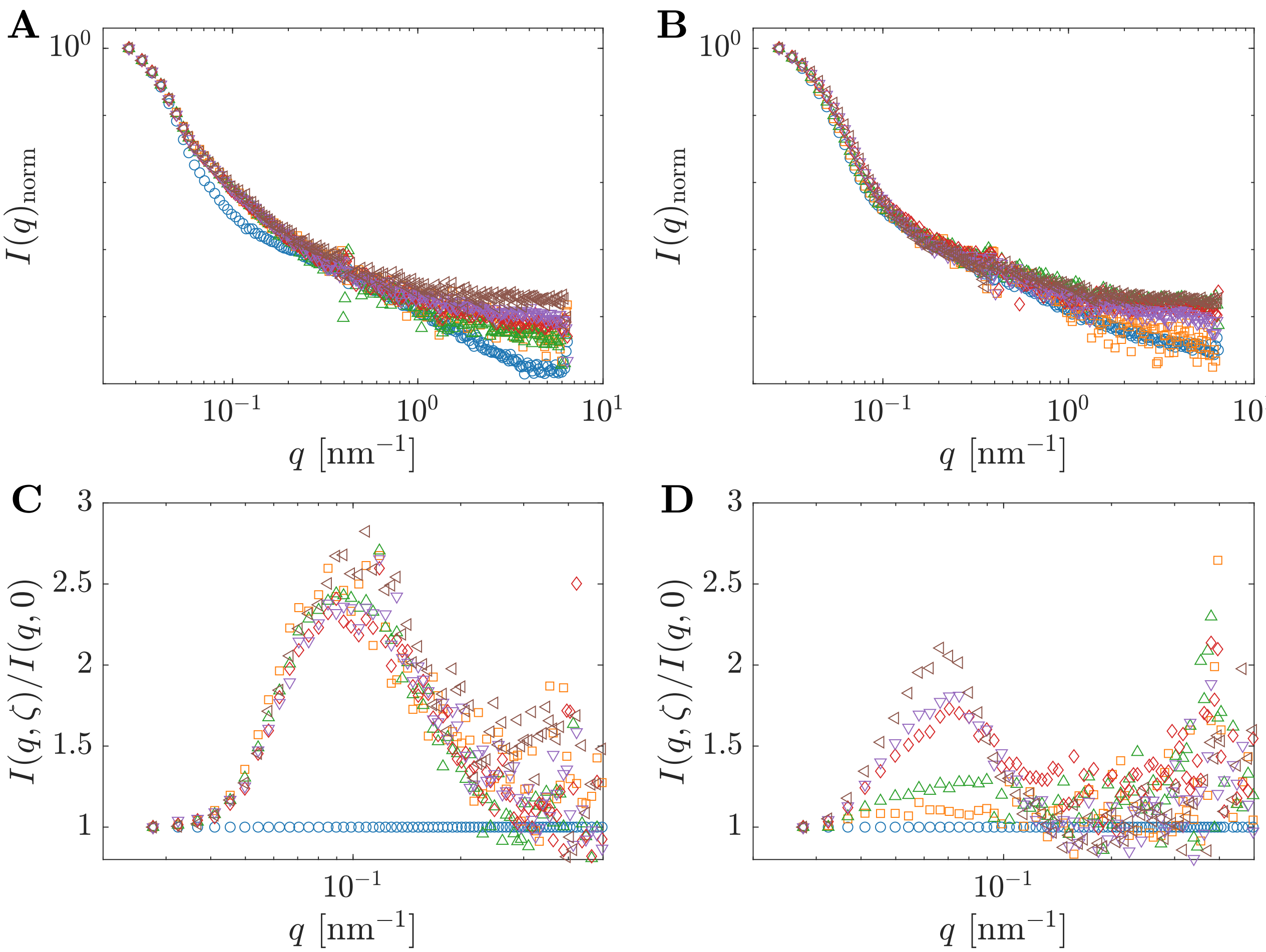

Figure S13: (**A,B**) SANS intensities normalized by the first datapoint $I(q)_{\mathrm{norm}}$ *vs.* scattering vector q for: (**A**) H_+ microgels in dilute state ($\zeta = 0.09 \pm 0.02$, blue circles) and in H_+/D_- mixtures at $\zeta = 0.33 \pm 0.03$ (orange squares), $\zeta = 0.76 \pm 0.06$ (green upward triangles), $\zeta = 1.04 \pm 0.09$ (red diamonds), $\zeta = 1.40 \pm 0.12$ (purple downward triangles) and $\zeta = 2.01 \pm 0.18$ (brown left triangles), or (**B**) H_- microgels in dilute state at $\zeta = 0.12 \pm 0.03$ (blue circles) and H_-/D_- mixtures at $\zeta = 0.31 \pm 0.03$ (orange squares), 0.74 ± 0.06 (green upward triangles), 1.54 ± 0.13 (red diamonds), 2.05 ± 0.18 (purple downward triangles), 3.06 ± 0.27 (brown left triangles). (**C,D**) Same data normalized by the SANS intensity at the lowest ζ for the H_+/D_- mixtures (**C**) and the H_-/D_- mixtures (**D**).

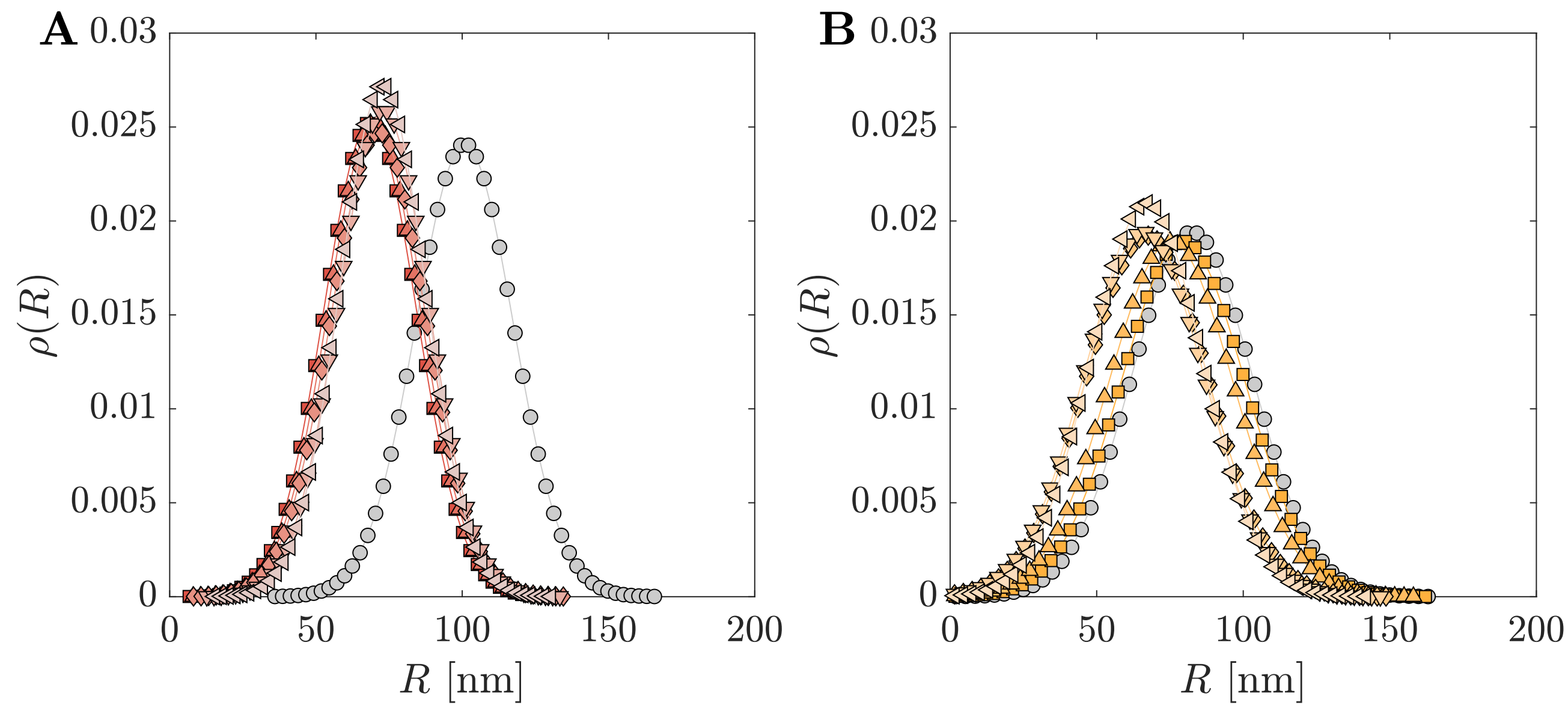

Figure S14: Size distributions $\rho(R)$ *vs.* R obtained from SANS data fits for: (**A**) H_+ microgels in dilute state ($\zeta = 0.09 \pm 0.02$, circles) and in H_+/D_- mixtures at $\zeta = 0.33 \pm 0.03$ (squares), $\zeta = 0.76 \pm 0.06$ (upward triangles), $\zeta = 1.04 \pm 0.09$ (diamonds), $\zeta = 1.40 \pm 0.12$ (downward triangles) and $\zeta = 2.01 \pm 0.18$ (left triangles), or (**B**) H_- microgels in dilute state at $\zeta = 0.12 \pm 0.03$ (circles) and H_-/D_- mixtures at $\zeta = 0.31 \pm 0.03$ (squares), 0.74 ± 0.06 (upward triangles), 1.54 ± 0.13 (diamonds), 2.05 ± 0.18 (downward triangles), 3.06 ± 0.27 (left triangles).

S7. Molecular dynamics simulations

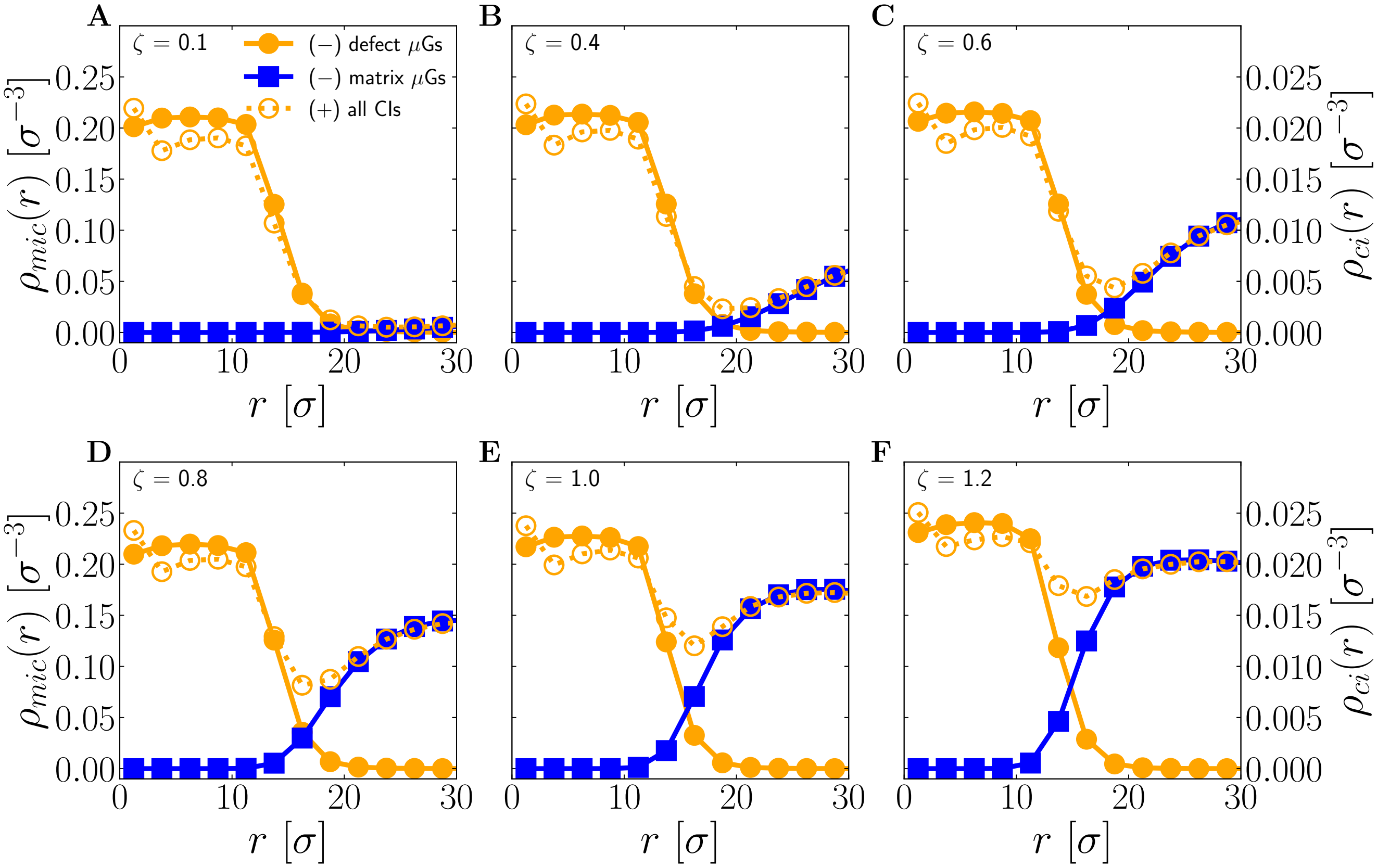

Figure S15: Simulated density profiles of negatively charged microgels in a matrix of negative microgels, (−/−), at varying volume fractions ζ (Indicated in the panels). Solid circles represent a central microgel, hollow circles are the corresponding counter-ions and squares denote the average density of the microgel matrix. For visibility the counter-ion density is scaled by a factor 10, which corresponds to the percentage of charged microgel beads.

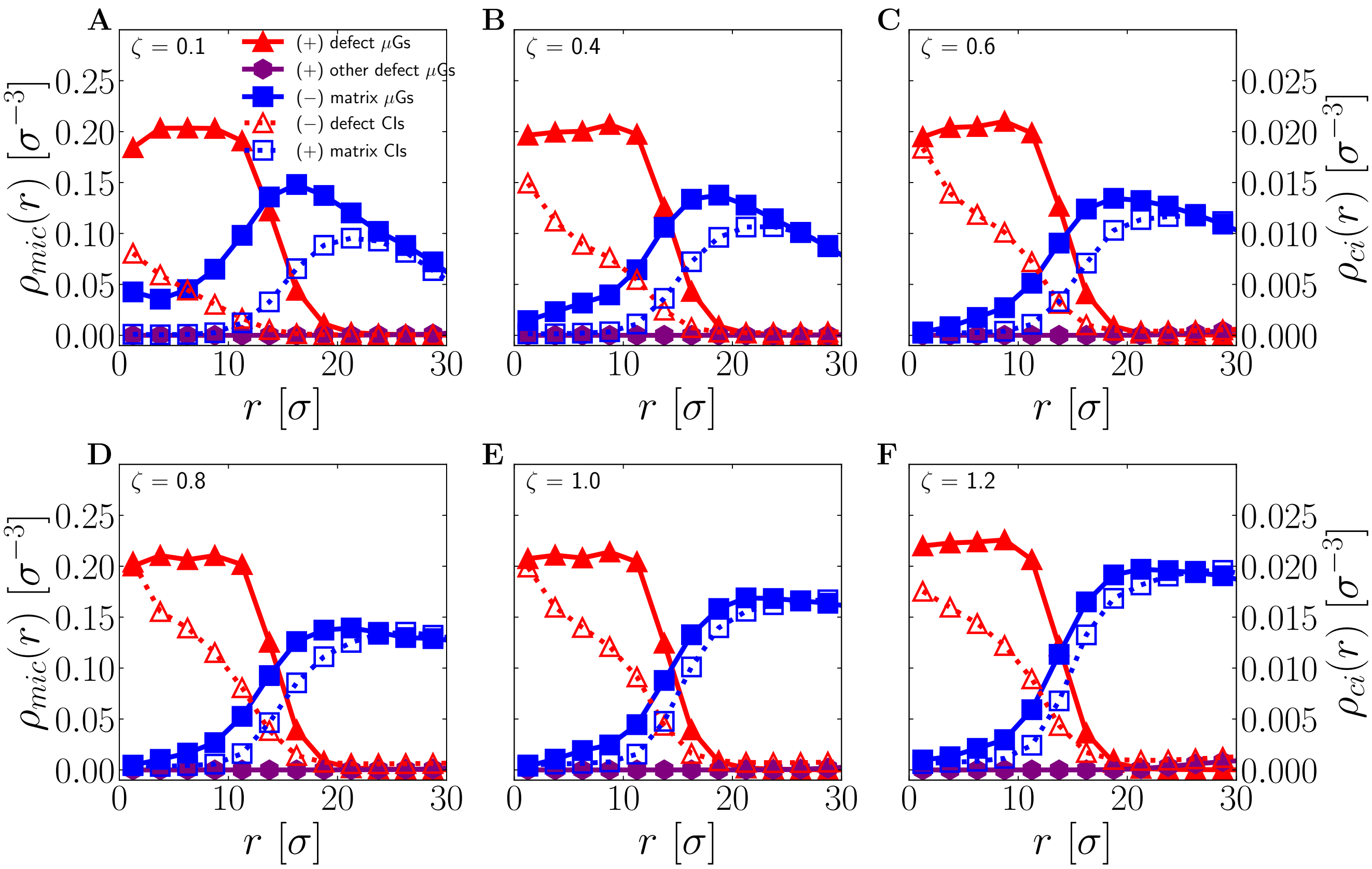

Figure S16: Simulated density profiles of positively charged microgels in a matrix of negative microgels, (+/–), at varying volume fractions ζ (Indicated in the panels). Solid upside triangles represent the central positively charged microgel, hollow upside triangles are the corresponding counter-ions, solid squares denote the average density of the negatively charged microgel matrix with its counter-ions being empty squares and the solid hexagons are other positively charged microgels in the matrix. For visibility the counter-ion density is scaled by a factor 10, which corresponds to the percentage of charged microgel beads.

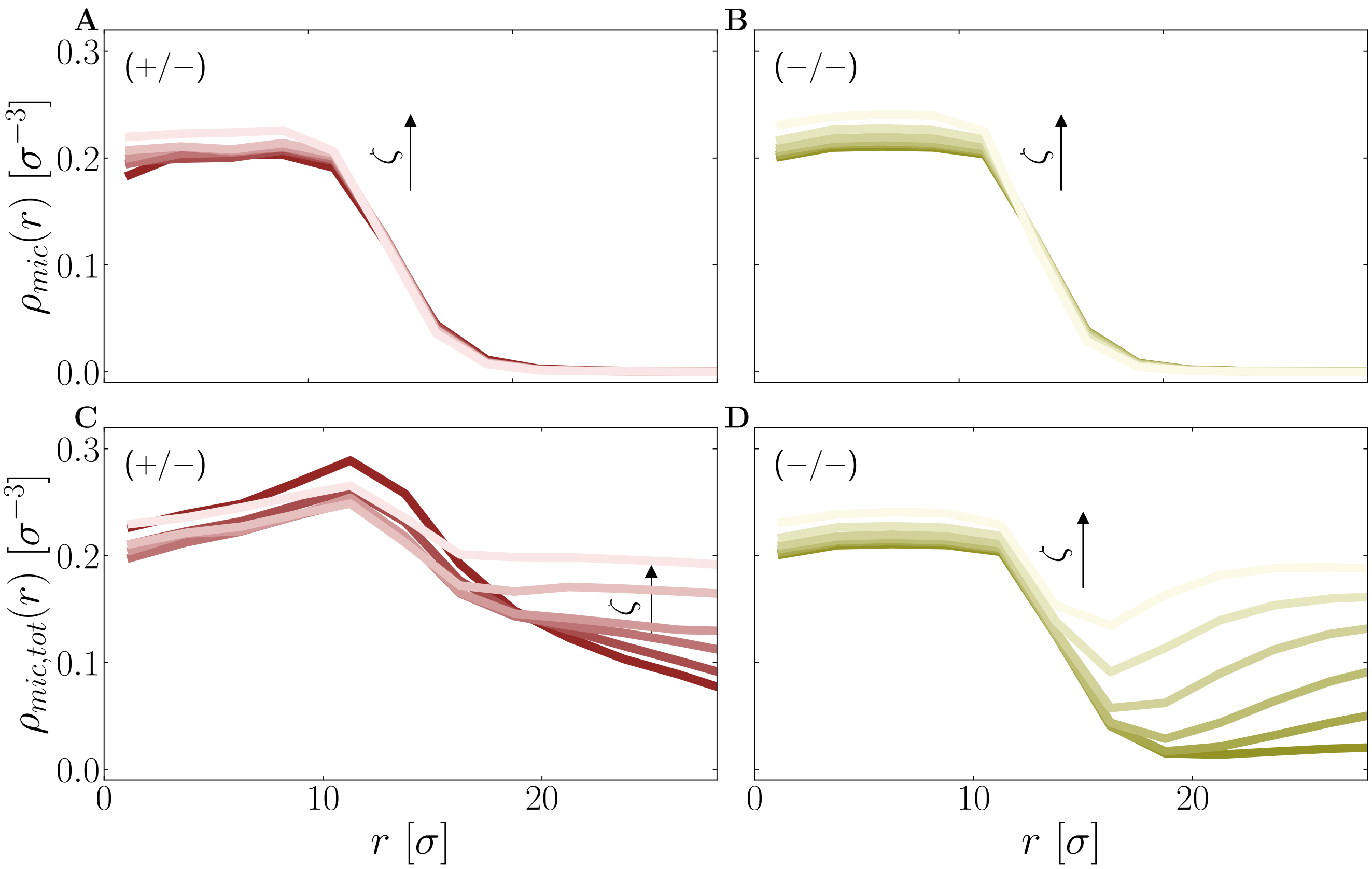

Figure S17: (**A**) Individual defect microgel density for the oppositely charged (+/−) mixture. (**B**) Individual defect microgel density for the similarly charged (−/−) mixture. (**C**) Total microgel density, combined from defect and matrix. for the oppositely charged (+/−) mixture. (**D**) Total microgel density for the similarly charged (−/−) mixture. Brighter colors denote higher generalized volume fractions

References

(1) Senff, H.; Richtering, W. Temperature sensitive microgel suspensions: Colloidal phase behavior and rheology of soft spheres. *The Journal of chemical physics* **1999**, *111*, 1705–1711.

(2) Romeo, G.; Imperiali, L.; Kim, J.-W.; Fernández-Nieves, A.; Weitz, D. A. Origin of de-swelling and dynamics of dense ionic microgel suspensions. *The Journal of Chemical Physics* **2012**, *136*.

(3) Batchelor, G. K. The effect of Brownian motion on the bulk stress in a suspension of spherical particles. *Journal of fluid mechanics* **1977**, *83*, 97–117.

(4) Scotti, A.; Gasser, U.; Herman, E.; Han, J.; Menzel, A.; Lyon, L. A.; Fernandez-Nieves, A. Phase behavior of binary and polydisperse suspensions of compressible microgels controlled by selective particle deswelling. *Physical Review E* **2017**, *96*, 032609.

(5) Cross, M. M. Rheology of non-Newtonian fluids: a new flow equation for pseudoplastic systems. *Journal of colloid science* **1965**, *20*, 417–437.

(6) Quemada, D. Rheology of concentrated disperse systems and minimum energy dissipation principle: I. Viscosity-concentration relationship. *Rheologica Acta* **1977**, *16*, 82–94.
